# 3D DNAzyme Motor Nanodevice With Self‐Powered FRET Amplifier and Self‐Supplied H_2_O_2_ for Enhancing Human Neutrophil Elastase Profiling and Chemodynamic Therapy in Lung Tumor

**DOI:** 10.1002/advs.202406599

**Published:** 2024-09-30

**Authors:** Huiyan Du, Ensheng Xu, Yihan Xu, Qingwang Xue, Hongxia Xu, Jibin Song

**Affiliations:** ^1^ Department of Chemistry Liaocheng University Liaocheng Shandong 252059 P. R. China; ^2^ State Key Laboratory of Chemical Resource Engineering College of Chemistry Beijing University of Chemical Technology Beijing 10010 P. R. China; ^3^ Department of Clinical Laboratory The Third People's Hospital of Liaocheng Liaocheng Shandong 252059 P. R. China

**Keywords:** 3D DNAzyme motor, chemodynamic therapy, self‐powered FRET amplifier, self‐supplied H_2_O_2_ HNE

## Abstract

The development of theragnostic nanosystems integrating FRET (fluorescence resonance energy transfer) imaging and chemodynamic therapy (CDT) for accurate diagnosis and effective treatment of lung tumors is still a big challenge. Herein, a peptide‐assembled 3D DNAzyme motor nanodevice is engineered for a self‐powered FRET amplifier profiling human neutrophil elastase (HNE) and self‐supplied H_2_O_2_ enhancing CDT. The nanodevice is prepared by depositing AuNPs on ZIF‐8, in which ZIF‐8 co‐loaded the lysosomal targeting peptide‐modified copper peroxides (PCPs) and hairpins (H1, H2, and H3), AuNPs are co‐labeled by DNAzyme‐peptide (DP) conjugate and H3. In the tumor micro‐environment, HNE driven 3D DNAzyme walker followed by an exponential amplification constructed by a synergistic cross‐activation between hybridization chain reaction and DNAzyme, generating a self‐powered FRET amplifier. The FRET amplifier specifically measures HNE with a sensitivity of 0.026 pM, and successfully images exogenous HNE in living cells and monitors HNE in mouse models. Moreover, the PCPs can target lysosomes, reducing lysosome escape. The self‐supplying H_2_O_2_ undertaken by PCPs improves the Cu (II)‐catalyzed Fenton‐like reaction, effectively causing cell apoptosis to inhibit tumor growth. Significantly, the nanodevice successfully screens inhibitors and discriminates the HNE level in normal and lung cancer tissues, suggesting that the nanodevice provides an effective tool for the diagnosis and treatment of lung tumors.

## Graphical abstract

A peptide‐assembled 3D DNAzyme motor nanodevice is engineered for a self‐powered FRET amplifier profiling human neutrophil elastase (HNE) and self‐supplied H_2_O_2_ enhancing chemodynamic therapy (CDT). The study demonstrates the advantages of 3D DNAzyme motor nanodevice in FRET imaging and CDT performance and provides an applicable tool for in vivo HNE detection and lung tumor diagnosis and treatment.

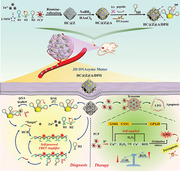

## 1 Introduction

Lung cancer is the leading cause of cancer‐related death worldwide due to late diagnosis, poor outcomes, and lack of efficient therapeutic modality.^[^ ^1^ ^]^ Accurate diagnose and efficient therapy of lung tumors at their earliest stages can significantly elevate the cure rate of cancer patients. Relevant biomarkers’ detection is of utmost importance for early clinic diagnosis and therapy of illnesses. Human neutrophil elastase (HNE) is an abundant serine protease that is a major constituent of lung elastolytic activity. Its aberrant expression contributes to the occurrence and progression of lung respiratory diseases, especially lung cancer.^[^ ^2^, ^3^ ^]^ Extensive studies showed that the level of HNE is highly involved in the acceleration of lung tumor growth and promotion of tumor invasion and metastasis through activating the PI3K‐AKT signal pathway.^[^ ^4^, ^5^ ^]^ Thus, HNE can act as a potential marker for the assessment of lung cancer risk and invasiveness, and a therapeutic target for drug discovery.^[^ ^6^, ^7^, ^8^ ^]^ Reliable and effective HNE analysis are crucial to early clinical diagnosis, targeted therapy, and prognosis monitoring of lung cancer. In situ, fluorescent sensing and imaging have attracted extensive attention because of their advantages of high sensitivity and spatial resolution.^[^ ^9^ ^]^ High‐resolution studies of HNE using activatable in situ fluorescence imaging may help to better understand changes in the activity and function of HNE, enabling real‐time monitoring of tumor treatment progress.^[^ ^10^ ^]^ To date, some fluorescent probes have been explored to in vivo image HNE and certified applicability for drug discovery and disease diagnosis.^[^ ^6^, ^11^, ^12^ ^]^ For example, a FRET‐system‐based nanoprobe by assembling CdSe/ZnS Quantum Dots (QD) was developed, which exhibited noninvasively measuring and imaging of HNE in vitro and in vivo.^[^ ^6^ ^]^ However, suffering from the limitation in the limit of detection (LOD), QD biosecurity, and organic molecular probes instability, their applications in vivo imaging of HNE remain a challenge for clinical diagnosis.^[^ ^13^, ^14^ ^]^ Consequently, it is urgent to develop a tool with the capability of high sensitivity, accuracy, and biosecurity for high spatial resolution imaging of HNE in living cells and in vivo.

Nucleic acids with intrinsic high biosecurity, excellent biocompatibility, ease of synthesis, and amenability have been regarded as perfect candidates for sensor arrays.^[^ ^15^, ^16^, ^17^ ^]^ Intelligent DNA molecular machines, which are specially designed components comprised of oligonucleotides based on the Watson‐Crick base pairing rule,^[^ ^18^, ^19^ ^]^ have been widely used in molecular imaging, biosensors, drug delivery due to remarkable signal amplification and logical assembly.^[^ ^19^, ^20^ ^]^ Notably, DNA walking devices, constructed using a walker, track, and energy input, show superior performance in biosensing and imaging due to their flexible programmability to design molecular walking behaviors with specific responses to different biological targets.^[^ ^21^, ^22^, ^23^, ^24^, ^25^ ^]^ In particular, the construction of a 3D DNA walking device demonstrates great potential for applications in biosensing, imaging, and material assembly due to the greater mobility and load capacity.^[^ ^26^, ^27^ ^]^ 3D DNA walking devices have become an effective tool for the imaging and tracking of biomolecules in living cells and in vivo, obtaining rich information concerning diseases. Gao et al. developed a 3D DNA walker device for Amyloid *β*‐peptide oligomer (A*β*O) detection and real‐time imaging in living cells and in vivo.^[^ ^18^ ^]^ As far as we know, there are currently rare reports that a 3D DNA walking device was designed for sensitive HNE detection and imaging in living cells and in vivo, obtaining abundant information concerning lung diseases. At present, one problem that restricts 3D DNA walking device In situ imaging of HNE *is* the amount of HNE in living cells and in vivo was below the detection threshold of effectively triggering the sensing probe in many cases, resulting in inefficient signal. Another problem is that lack of the target conversion limited the DNA walking machine for HNE sensing and imaging. Thus, developing a versatile 3D DNA walking device that is integrated with other amplification means is necessary for achieving an improved sensing performance in HNE detection and real‐time imaging in living cells and in vivo.

Theragnostic systems that enable diagnosis, therapy, and monitoring of therapeutic response at the same time have drawn the growing attention of researchers. At present, chemodynamic therapy (CDT), which produces highly toxic hydroxyl radicals (•OH) by activating the Fenton or Fenton‐like reaction in tumors to kill tumor cells, has gained popularity due to their non‐invasive characteristics, in situ ROS generation, and deep penetration.^[^ ^28^, ^29^, ^30^, ^31^, ^32^, ^33^ ^]^ However, CDT that uses endogenous H_2_O_2_ to produce toxic reactive oxygen species (ROS) for killing cancer cells is limited by unsatisfactory efficacy due to the insufficient intracellular H_2_O_2_ level.^[^ ^34^ ^]^ Much research effort is being devoted to enhancing the efficiency of CDT. Notably, introducing H_2_O_2_‐supplementing functionality into CDT agents is a feasible alternative method for enhancing CDT. In particular, metal peroxide nanoparticles have emerged as promising H_2_O_2_ sources for CDT nanoagents.^[^ ^29^, ^35^, ^36^ ^]^ For example, Chen et al synthesized copper peroxide (CP) nanodots as an activatable chemodynamic agent for enhanced CDT by self‐supplying H_2_O_2_. The CP nanoparticles that served as the chemodynamic agent produce ROS to kill cancer cells by disrupting lysosomal membrane integrity and triggering lysosomal lipid peroxidation (LPO).^[^ ^29^ ^]^ Targeting lysosomal membrane permeability and integrity is one of the anti‐tumor strategies in targeting lysosomes.^[^ ^37^ ^]^ Increasing evidence reveals that selective delivery of drugs/nanoagents to specific subcellular organelles can significantly enhance the efficiency of cancer therapy.^[^ ^38^ ^]^ The CP nanoparticles that were used as the chemodynamic agent for enhanced CDT confronted with the discounted therapeutic effect owing to the lack of lysosomal targeting and the lysosome escape, thereby resulting in a low level of ROS in lysosomal subcellular organelles, and increasing the oxidative stress resistance of cancer cells. Consequently, how to improve CP delivery to lysosomes for enhancing local ROS levels and cancer treatment is a challenge in CP‐enhanced CDT.

Developing an all‐in‐one nanosystem that is capable of sensitive sensing, imaging in living cells and in vivo, and high‐efficiency CDT will be considerably valuable for dynamic monitoring HNE and effective treatment in lung tumors. Herein, we engineered a peptide‐assembled 3D DNAzyme motor nanodevice for a self‐powered FRET amplifier profiling lung tumor‐related HNE and for self‐supplied H_2_O_2_ enhancing CDT. The nanodevice is prepared by depositing Au nanoparticles on ZIF‐8, in which ZIF‐8 co‐loaded the lysosomal targeting peptide modified copper peroxide (PCP) and hairpins (H1, H2, and H3), Au nanoparticles were co‐labeled by DNAzyme‐peptide (DP) walking strands and H3 tracks. The nanodevice, also termed HC@Z@A/DPH nanocomposites, served dual roles in chemodynamic tumor therapy and in situ FRET monitoring of HNE. In the tumor micro‐environment, the nanodevice was degraded, releasing the hairpins, PCP nanodots, and the assembled 3D DNAzyme walker that was constructed of AuNP, H3 track, and DNAzyme‐peptide (DP) walking strands. On the one hand, the target HNE recognized DP walking strands and activated the 3D DNAzyme walker followed by an enzyme‐free exponential amplification (EFEA) constructed by the synergistic cross‐activation between hybridization chain reaction (HCR) and DNAzyme, resulting in the repeated formation of the long DNA concatamer to generate a self‐powered FRET amplifier. The DP probe converts HNE to DNA, which can be highly amplified by the self‐powered FRET amplifier. The self‐powered FRET amplifier enhanced fluorescence, improving sensitivity and accuracy for imaging of HNE. On the other hand, the nanodevice as the nanocarrier increases the PCP loading and improves the Cu(II)‐catalyzed Fenton‐like reaction. The self‐supplying H_2_O_2_ undertaken by PCPs promoted ROS (•OH) generation and inactivated GPX4, enhancing LPO accumulation and CDT. Additionally, the PCP nanoparticles could target the lysosome of tumor cells through lysosomal targeted peptide, reducing lysosome escape, enabling high tumor accumulation inhibiting tumor growth, and down‐regulating the HNE expression. Significantly, the nanodevice is capable of screening potential inhibitors and discriminating the HNE level in normal person and lung cancer patient tissues, suggesting that the nanodevice provides an effective tool for accurate diagnosis and treatment of lung tumors.

## 2 Results and Discussion

### 2.1 Principle of 3D DNAzyme Motor Nanodevice for Enhancing HNE Profiling and Chemodynamic Therapy in Lung Tumor

**Scheme 1:**
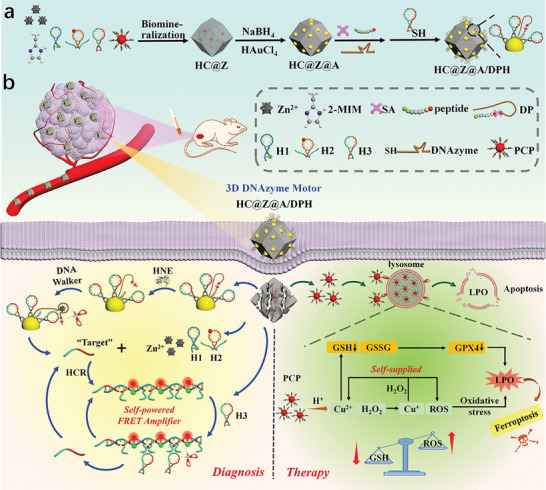
Schematic Diagram of 3D DNAzyme motor nanodevice with self‐powered FRET amplifier and self‐supplied H_2_O_2_ for enhancing HNE profiling and chemodynamic therapy in lung tumor. a) Schematic illustration of the synthesis route of the 3D DNAzyme motor nanodevice HC@Z@A/DPH nanocomposites. b) 3D DNAzyme motor nanodevice HC@Z@A/DPH nanocomposites for in vivo FRET imaging of HNE and chemodynamic therapy in lung tumors.

The working principle of 3D DNAzyme motor nanodevice is illustrated in **Scheme** **1**. In the 3D DNAzyme motor nanodevice, the lysosomal targeting peptide‐modified copper peroxide (PCP) and multiple hairpins (H1, H2, and H3) were first co‐encapsulated in ZIF‐8 through in situ biomineralization, being called hairpins&PCPs@ZIF‐8 (HC@Z for short). Here H1 and H2 were modified by Cy3 and Cy5 respectively, and a split sequence of Zn^2+^‐dependent DNAzyme was ingeniously designed at both ends of H2. H3 contained a mimic sequence similar to “target” as the initiator that can initiate HCR. Subsequently, Au nanoparticles with uniform grain distribution were directly grown on the surface of HC@Z through in situ reduction, called HC@Z@A. One end of the HNE‐specific peptide was anchored to the surface of Au nanoparticles by sulfhydryl group, and the other end was connected with the DNAzyme walking strand with a specific sequence of Zn^2+^‐dependent DNAzyme by biotin‐streptavidin conjugation, in which another end of DNAzyme walking strand was also anchored to the surface of the Au nanoparticles by sulfhydryl group. When DNAzyme‐peptide (DP) conjugate was immobilized, the H3, meanwhile, was also fixed on the Au nanoparticle, forming the hairpins&PCPs@ZIF‐8@Au/DP&H3 (HC@Z@A/DPH for short) nanocomposites. In the tumor microenvironment, HC@Z@A/DPH nanocomposites (termed 3D DNAzyme motor nanodevice) were degraded, releasing the hairpins (H1, H2, H3), PCP, and the assembled 3D DNAzyme walker constructed of AuNP, H3 track, DP walking strands. The 3D DNAzyme motor was activated by the 3D DNAzyme walker when target HNE recognized and cleaved the DP conjugate and DNAzyme walking strand was unblocked. The autonomous walking was driven by Zn^2+^ from ZIF‐8, cleaving the tracks H3 on AuNP, and releasing a large number of “target” DNA as primer for HCR. Through HCR reaction, the liberated H1 and H2 from ZIF‐8 was assembled, forming of tandem DNAzyme nanowires nanostructures, and bringing the FRET pair into close proximity and inducing an amplified FRET signal. At the time, the HCR‐assembled tandem DNAzyme nanowires served as biocatalysts to cleave the liberated H3 substrate from ZIF‐8, producing numerous new triggers for reversely stimulating HCR amplifier and further realizing the exponential amplification to generate a self‐powered FRET amplifier. The self‐powered FRET amplifier enhanced fluorescence, improving sensitivity and accuracy for imaging of HNE. Simultaneously, the released PCPs could target the lysosome of tumor cells through lysosomal targeted peptide, reducing lysosome escape, causing high tumor accumulation, and amplified local oxidative stress. In the lysosome of the tumor cell, the PCPs decompose, producing Cu^2+^ and H_2_O_2_. The Fenton‐like reaction between Cu^2+^ and H_2_O_2_ generates ROS (•OH) and Cu^+^, and that between Cu^+^ and H_2_O_2_ generates ROS and Cu^2+^, forming a self‐supplying cyclic catalysis effect. Cu^2+^ reacts with glutathione (GSH) to generate Cu^+^ and GSSG. Simultaneously, the GSH consumption decreases the glutathione peroxidase 4 (GPX4) expression. The generated ROS (•OH) induced lysosomal lipid peroxidation (LPO) of the lysosomal membrane, resulting in lysosomal membrane permeabilization (LMP) and cell apoptosis, effectively inhibiting tumor growth and down‐regulating the HNE expression. The nanodevice as the nanocarrier increases the PCP loading and improves the Cu(II)‐catalyzed Fenton‐like reaction. More notably, the 3D DNAzyme motor nanodevice is capable of screening potential inhibitors and discriminating the HNE expression in normal person tissues and lung cancer patient tissues, suggesting that the nanodevice provides an effective tool for accurate diagnosis and treatment of lung tumors.

### 2.2 Characterization of CPs and HC@Z@A/DPH Probe

**Figure 1:**
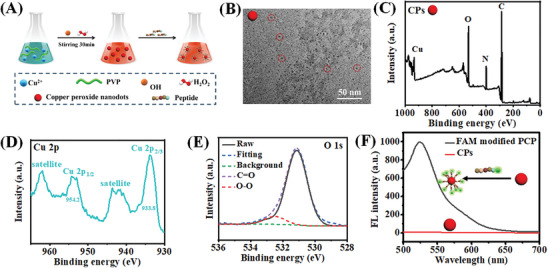
Preparation and characterization of the CPs. A) Illustration of CPs preparation, B) TEM image of CPs, C) Full range XPS spectra, D, E) The corresponding high‐resolution XPS spectra of Cu 2p and O 1s for CPs. F) Fluorescence emission spectra of CPs, and CPs functionalized by targeting peptide with FAM fluorophore.

The preparation of CPs is illustrated in **Figure** **1**A. TEM images exhibited that the CPs were ≈6 nm with good dispersibility (Figure S1A, Supporting Information). Irregular and cloud‐like substances were in the surrounding area of CPs, which was probably caused by the PVP decoration (Figure 1B). The PVP decoration was characterized by FT‐IR and XPS (Figure 1C; Figure S1C, Supporting Information). Two absorption bands ≈1652 and 1293 cm^−1^ can characterize stretching vibrations of C = O and C−N bonds in PVP respectively, indicating the decoration of PVP on CPs (Figure S1C, Supporting Information). Besides, the wide scan XPS spectra support the presence of C, O, N, and Cu, which further demonstrates the presence of PVP along with CPs (Figure 1C). What's more, the Cu 2p_1/2_ and Cu 2p_3/2_ peaks in CPs were centered at 953.6 and 934.5 eV well‐distinguishable dominant peaks together with two satellite peaks at 962.4 and 943.1 eV respectively, which showed the formation of CuO_2_ with the oxidation state Cu (+2) (Figure 1D). Furthermore, two peaks with binding energies 530.7 and 532.5 eV are characteristic of C = O and O−O (Figure 1E), indicating the presence of PVP and peroxo groups in CPs. Meanwhile, UV–Vis spectra were employed to distinguish the synthesized CPs from copper oxide nanoparticles (CuO NPs) (Figure S1B, Supporting Information), which further verified the successful synthesis of CPs. To demonstrate the lysosomal targeting peptide successfully modified on the CPs, we chose a targeting peptide with fluorophore (Figure 1F). The increase in fluorescence intensity after the biofunctionalization indicated the successful functionalization of CPs with peptides.

**Figure 2:**
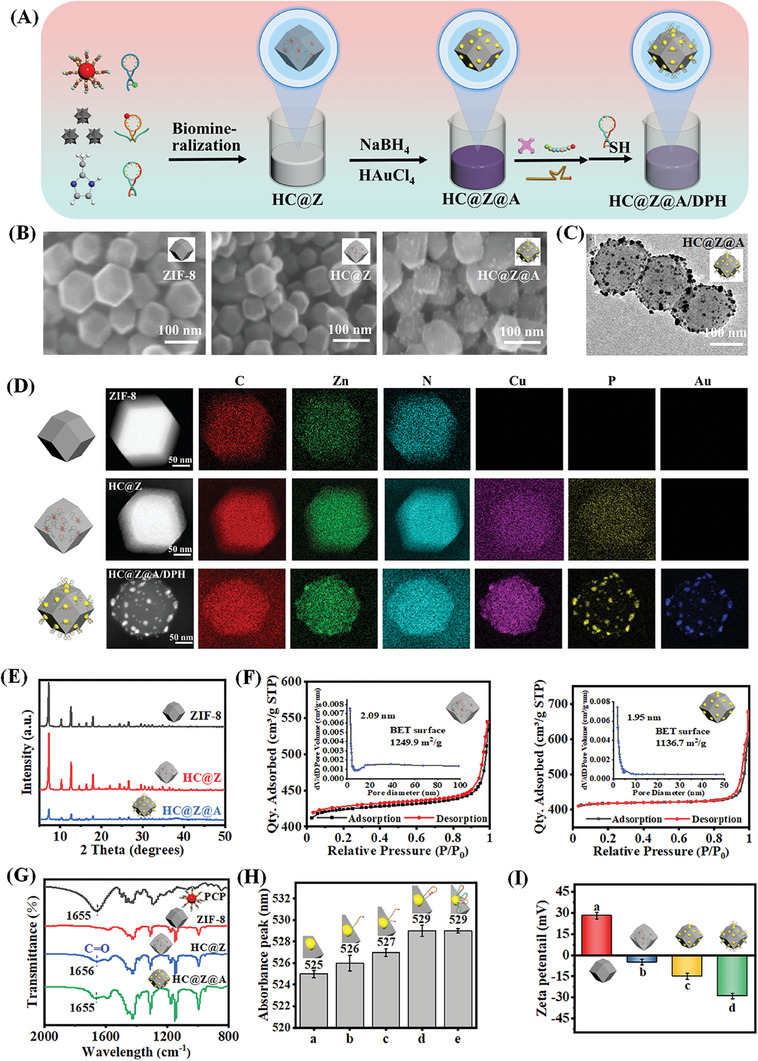
Characterization of 3D DNAzyme motor nanodevice. A) Synthesis procedure of 3D DNAzyme motor nanodevice (HC@Z@A/DPH nanocomposites). B) SEM image of ZIF‐8, hairpins&PCPs@ZIF‐8 (HC@Z), hairpins&PCPs@ZIF‐8@Au (HC@Z@A). C) TEM image of HC@Z@A. D) HAADF‐STEM image and EDS elemental maps of ZIF‐8, HC@Z, HC@Z@A/DPH. **E)** XRD patterns of ZIF, HC@Z, and HC@Z@A. F) Nitrogen adsorption‐desorption isotherm at 77 K, and DFT pore size distribution with N_2_ at 77 K of HC@Z (left) and HC@Z@A (right). G) FTIR spectrum of PCPs, ZIF‐8, HC@Z, and HC@Z@A. H) Shift of the UV–Vis absorbance peaks with the AuNP in each nanomaterial (a‐d: HC@Z@A, HC@Z@A/D, HC@Z@A/D/P, HC@Z@A/DP, HC@Z@A/DPH). I) Zeta potential of ZIF‐8 (a), HC@Z (b), HC@Z@A (c), and HC@Z@A/DPH probe.

In this study, the 3D DNAzyme motor nanodevice (HC@Z@A/DPH nanocomposites) were prepared by loading equal amounts of hairpins (H1, H2, and H3) and PCPs on the ZIF‐8, and in situ growing AuNPs on ZIF‐8 and functionalizing AuNPs with H3 and DNAzyme‐peptide (DP) conjugates (**Figure** **2**A). First, the morphologies of ZIF‐8, hairpins&PCPs@ZIF‐8 (HC@Z), hairpins&PCPs@ZIF‐8@Au (HC@Z@A) was characterized by SEM (Figure 2B), which exhibited a cubic, typical rhombic dodecahedron. SEM images confirmed that the embeddedness of PCPs and hairpins into ZIF‐8 and the in situ growing AuNPs on ZIF‐8 appear to have no significant impact on the framework structure of ZIF‐8, which has also been verified by XRD (Figure 2E). HAADF‐STEM images further elucidate the formation of HC@Z (Figure 2D). The elemental mapping verified the elements of C, N, O, Zn, Cu, and P in HC@Z nanocomposites, meanwhile exhibiting the uniform distribution of the Cu and P elements from copper peroxide and hairpins inside HC@Z. Additionally, EDS elemental line scanning spectra of Zn, Cu, and P elements in hairpins&PCPs@ZIF‐8 (HC@Z) and PCPs/ZIF‐8/DNA further certify that PCPs and hairpins were encapsulated inside the pore of ZIF‐8, rather than physically adsorbed onto the surface of ZIF‐8 (Figure S2A, Supporting Information). It was observed that Zn elements are uniformly distributed throughout the hairpins&PCPs@ZIF‐8, Cu and P elements increased as the distance from the nanocomposites center reduced small, implying the large number of PCPs and hairpins inside the pore of ZIF‐8. By comparison, the Cu and P contents in PCPs/ZIF‐8/DNA were uniformly distributed over the whole surface of ZIF‐8, which showed a statistically significant difference compared to that of hairpins&PCPs@ZIF‐8 (HC@Z). Besides that, we determined the average content of Cu in HC@Z to be ≈2.13 wt.% by ICP‐MS (Table S2, Supporting Information), which could be converted into 3.20 wt.% of the PCP content. Hairpins encapsulation efficiency is as high as 97.7% by using fluorescence detection after labeling them (Figure S2C, Supporting Information), corresponding to 4.7 × 10^14^ hairpins per µg of hairpins&PCPs@ZIF‐8 (HC@Z). After the HC@Z in situ growing of AuNPs, gold nanoparticles homogeneously dispersed on HC@Z surface with a rough surface and had no noteworthy influence on the morphology and diameter of ZIF‐8 (Figure 2B). The TEM observations revealed that Au nanoparticles are uniformly distributed on the HC@Z surface, and the average particle size is ≈3 nm, with several uneven agglomerated particles (Figure 2C). From the HRTEM (Figure S2B, Supporting Information), we observed that the HR‐TEM image shows clear lattice fringes with d‐spacing values of ≈0.232 and ≈0.207 nm corresponding to the (111) and (200) lattice planes, respectively, indicating the crystalline nature of Au. Meanwhile, we also observed the Au element in the elemental mapping of hairpins&PCPs@ZIF@Au (HC@Z@A) HAADF‐STEM image (Figure 2D). The aforementioned results are capable of demonstrating the successful formation of HC@Z@A. As seen from Figure 2E, XRD showed the absence of characteristic peaks for the in situ grown Au NPs, indicating that the in situ grown AuNPs did not destroy the ZIF‐8 structure. Moreover, the encapsulation of PCPs in HC@Z@A was also verified by UV–Vis and FT‐IR spectroscopy (Figure 2G; Figure S2D, Supporting Information). Thermogravimetric analysis (TGA) further confirmed the presence of hairpins, PCPs, and Au in HC@Z@A nanocomposites (Figure S2E, Supporting Information). Besides that, N_2_ adsorption‐desorption isotherms indicated the size distribution of the porous structure in HC@Z and HC@Z@A (Figure 2F), which presented a high BET surface area of 1249.9, 1136.7 m^2^ g^−1^ and an average pore diameter of 2.09 and 1.95 nm respectively, and exhibited type I isotherms. As anticipated, the Au nanoparticles just grew on the external surface of HC@Z and not embedded in the pores, and exhibited the characteristic of microporous materials. Thus, the high surface area is capable of encapsulating more PCPs and hairpins in HC@Z@A.

To construct 3D DNAzyme motor nanodevice, the H3 track and DP conjugate walking strand were assembled on the HC@Z@A. UV–Vis absorption of each nanomaterial (HC@Z@A, HC@Z@A/D, HC@Z@D/P, HC@Z@A/DP, HC@Z@A/DPH) was performed to certify the functionalization of AuNPs in HC@Z@A through the absorbance peak shift.^[^ ^39^ ^]^ This peak shifted toward the longer wavelength as some biomaterials were attached to the surface of the AuNP in HC@Z@A due to the LSPR effect.^[^ ^40^ ^]^ This UV absorbance method was suitable for directly recognizing binding events between biomolecules and nanomaterial. As seen from Figure 2H, the generated AuNPs in HC@Z exhibited the absorbance peak at 525 nm, whereas the bifunctional AuNP absorption peak of the UV–Vis spectrum gradually red‐shifted by ≈1–4 nm. The changes in the absorbance peak could be used to successfully evaluate the linkage of DNAzyme‐peptide (DP) conjugate and hairpin H3 to the AuNPs in HC@Z@A. In addition, the DLS results revealed the HC@Z@A/DPH composites possessed a bigger size distribution than HC@Z@A (Figure S3A, Supporting Information). As shown in Figure 2I, the zeta potential changed from positive values (+27.2 mV) for ZIF‐8 to negative ones (−10.9 mV) when hairpins (H1, H2, and H3) and PCPs were encapsulated into ZIF‐8. DNA is negatively charged, which caused the change of zeta potential of HC@Z. The zeta potential became more negative when Au nanoparticles were grown on the surface of HC@Z through in situ reduction. Interestingly, the zeta potential of HC@Z@A/DPH becomes more negative as the tracking strand (H3) and walking strand (DP) were assembled on the HC@Z@A, which demonstrated the successful formation of HC@Z@A/DPH (also termed 3D DNAzyme motor nanodevice). Furthermore, elemental mapping in the HAADF‐STEM image further identifies the assembly of DNA and peptides in HC@Z@A (Figure 2D). Seen from Figure 2D and P element from DNA could be observed in the AuNP of HC@Z@A nanocomposites, indirectly indicating the HC@Z@A conjugate with H3 track and DP conjugate walking strand. Meanwhile, EDS displayed the relative amount of elements Au, C, N, P, Cu, and Zn (Figure S3B, Supporting Information). Moreover, the elemental and valence composition were analyzed by XPS (Figure S3C–H, Supporting Information). The XPS survey spectrum confirmed the existence of the Au, C, N, P, Cu, and Zn in the HC@Z@A/DPH nanocomposites, further verifying the successful preparation of the HC@Z@A/DPH. According to the calculation, every µg of HC@Z was averagely coated by 120 ng of AuNPs, there were ≈2.5 × 10^11^ the H3 track and walking 0.85 × 10^9^ DP per ng of AuNPs on the basis of the fluorescence standard curve, respectively.

### 2.3 Feasibility of the DNA Walker Cascaded EFEA Amplification Strategy and 3D DNAzyme Motor Nanodevice for HNE Detection

**Figure 3:**
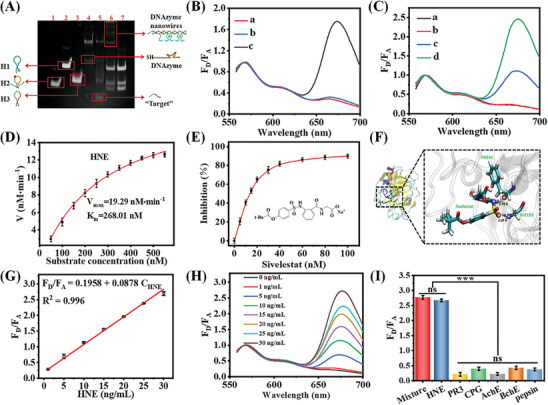
A) Polyacrylamide gel electrophoresis demonstration of the DNAzyme cleavage and HCR reaction.(B) Fluorescence spectra of the 3D DNAzyme motor nanodevice HC@Z@A/DPH without HNE (a), the hairpins&PCPs@ZIF‐8@Au/H3 with HNE (b), the complete probe of H2.1 with HNE (c) and the 3D DNAzyme motor nanodevice HC@Z@A/DPH nanocomposites with HNE (d). C) Fluorescence spectra of the 3D DNAzyme motor nanodevice HC@Z@A/DPH without HNE under pH 5.5 (a), with HNE under pH 7.4 (b), and with HNE under pH 5.5 (c). D) Measurement of initial velocity versus the HC@Z@A/DPH nanocomposites concentration for kinetic analysis (HNE 0.1 µg mL^−1^). E) Inhibition effect of Sivelestat toward HNE activity with the 3D DNAzyme motor nanodevice HC@Z@A/DPH. F) The molecular modeling of HNE‐Sivelestat. Amino acid chains represented HNE proteins in solid color bands, ball‐and‐stick models are representative of Sivelestat, and black dashed lines are representative of hydrogen bonds. G) Fluorescence spectra of 3D DNAzyme motor nanodevice HC@Z@A/DPH in response to different concentrations of HNE. The reactions were performed with HNE from 1 to 30 ng mL^−1^. H) The linear relationship of the HNE concentration and the fluorescence intensity. I) Specificity profile of HC@Z@A/DPH nanocomposites toward 25 ng mL^−1^ HNE, PR3 (proteinase 3), CPG (cathepsin G), and BChE (butyrylcholinesterase), AChE (acetylcholinesterase), pepsin, a mixture of HNE and other proteases. ns, no significance, ***p < 0.001.

Nondenaturating polyacrylamide gel electrophoresis (PAGE) was performed to examine the feasibility of the DNA walker cascaded EFEA amplification strategy. As shown in **Figure** **3**A, the bands in lanes 1, 2, and 3 correspond to H1, H2, and H3 respectively. Lane 4 represents the DNAzyme strand, in which the high‐molecular‐weight band was attributed to the disulfide bond in DNAzyme. After co‐incubating DNAzyme with H3, a new low‐molecular‐weight band was found accompanied by H3 bands becoming darker (Lane 5), indicating the DNAzyme cleavage reaction occurring, and indirectly verifying the DNA walker feasibility. When all strands of H1, H2, H3, and DNAzyme were co‐incubated, a well‐defined band of amplified products is observed along with H1, H2, and H3 bands becoming darker. The large molecular weight of amplified products was mainly due to the DNAzyme cleavage reaction between DNAzyme and H3‐initiated HCR to form wire‐shaped DNA nanostructures (Lane 6). In contrast, no band of amplified products is observed in the control group without DNAzyme strand (Lane 7), indicating the failure of HCR, and DNAzyme cleavage reaction between DNAzyme and H3. The results indirectly demonstrate that the DNA walker cascaded HCR strategy can be well performed. Additionally, we investigate the EFEA reaction using gel electrophoresis analysis (Figure S4A, Supporting Information). The EFEA reaction was achieved by exploiting the synergistic cross‐activation between HCR and DNAzyme. The presence of H3 yielded larger amounts of DNA nanostructure product, and H3 bands became darker compared with lane 1 (Lane 2), indicating that HCR‐assembled DNAzyme can cleave H3 and EFEA reaction occurs. In contrast, no larger amounts of DNA nanostructure product bands are observed in the absence of H3 compared with lane 2, indicating no occurrence of EFEA reaction (Lane 3). The above results show that the designed DNA walker cascaded EFEA amplification strategy can be successfully performed.

We examined the morphology of HC@Z@A/DPH nanocomposites incubated in Tris‐HCl buffer at different pH (7.4, and 5.5). As seen from Figure S4B (Supporting Information), HC@Z@A/DPH nanocomposites exhibited good stability in a physiological environment (pH 7.4) but obviously degraded in acid conditions corresponding to lysosomes(pH 5.5). Next, ICP‐MS was employed to determine the released Zn^2+^ from HC@Z@A/DPH nanocomposites. Notably, we observed that Zn^2+^ content was significantly higher than in controls (pH 7.4) (Figure S4C, Supporting Information), in which Zn^2+^ concentration was hardly obvious changes. Besides, the release studies were performed under various pH and time conditions (Figure S4D,E, Supporting Information). Our results indicate the cumulative release rate of hairpins and PCPs for pH 5.5 (tumor micro‐environment) was higher than that for pH 7.4 (physiological environment). The cumulative release reached 80% within 2 h at pH 5.5, staying consistent with the lysosome environment, indicating a high responsivity of HC@Z@A/DPH nanocomposites to an acidic tumor micro‐environment.

Subsequently, we verify the implement of the designed 3D DNAzyme motor nanodevice HC@Z@A/DPH for HNE under different conditions in vitro by fluorescence spectroscopic analysis (Figure 3B). As shown in Figure 3B, there is only a very low FRET signal (F_D_/F_A_) when no HNE was added into the HC@Z@A/DPH, implying that no DNA walker and EFEA reaction occurred (line a). In the presence of HNE, the hairpins&PCPs@ZIF‐8@Au/H3 (HC@Z@A/H) also exhibited very low FRET that was close to that of the blank group, indicating that no DNA walker and EFEA occurred (line b). In contrast, there was a 2.4‐fold enhancement in the FRET when HNE was incubated with 3D DNAzyme motor nanodevice HC@Z@A/DPH, indicating a successful DNA walker and EFEA reaction (line d). This could be attributed to the HCR‐assembled DNAzyme nanowires cleaving the released H3 substrate form ZIF‐8, producing numerous new triggers for reversely stimulating HCR amplifier and further realizing the exponential amplification for the FRET signal enhancement. To demonstrate the enhanced FRET signal derived from the cleavage of H3 with mimetic sequence, H2.1 without the split DNAzyme sequence was designed as a control (line c). One observed that a lower FRET signal was obtained for H2.1, which offers direct proof that the DNA walker cascaded EFEA strategy is more sensitive than the traditional DNA walker‐HCR. Next, to verify the 3D DNAzyme motor nanodevice HC@Z@A/DPH for sensitive detection of HNE, the fluorescence responses of the HC@Z@A/DPH nanocomposites toward HNE were surveyed under different conditions of pH (7.4 and 5.5), simulating physiological and lysosome conditions. As seen from Figure 3C, the HC@Z@A/DPH nanocomposites were stable in the absence of HNE under acidic conditions, and no obvious fluorescence changes were detected under physiological conditions with the target, owing to the confinement of DNAzyme walk stand, and lack of cofactors as well as hairpin probes for DNA walker‐EFEA cascade amplification (line a, b). In contrast, there was a high fluorescence response under acidic conditions with target HNE (line c). This is because the HC@Z@A/DPH nanocomposites were degraded under acidic conditions, liberating hairpin probes and releasing Zn^2+^ cofactors for DNA walker cascaded EFEA amplification in the presence of target HNE. The results confirmed that the 3D DNAzyme motor nanodevice HC@Z@A/DPH could be used for FRET detection of HNE through DNA walker cascaded EFEA amplifier under an acidic tumor micro‐environment.

### 2.4 Enzyme Kinetics and Inhibition Characterization

To determine the initial rate of HNE reaction, we first derived a standard curve by measuring the FRET after different amounts of HC@Z@A/DPH nanocomposites are fully involved in the reaction (Figure S5A, Supporting Information). When measuring enzyme kinetics, a certain amount of HNE was added to HC@Z@A/DPH nanocomposites with different concentrations for 10 min and then inactivated the enzyme. Subsequently, the HC@Z@A/DPH nanocomposites itself was subjected to the following amplification reaction to obtain the FRET to determine the actual substrate amounts consumed. The enzymatic reaction obeys the Michaelis‐Menten mechanism, and the values of the kinetic parameter, Michaelis constant *Km*, and maximum velocity *Vmax*, are estimated.

(1) $$v=\frac{d\left[P\right]}{dt}=\frac{V_{\max}\left[S\right]}{K_{m}+\left[S\right]}$$

We investigated the kinetic characterization of HNE activity and its inhibitors with HC@Z@A/DPH nanocomposites as substrate. The time‐dependent FRET response was quantified in Tris‐HCl (15 mM, pH 5.5) with HNE and in the presence of different concentrations of HC@Z@A/DPH nanocomposites substrate. The initial hydrolysis velocity diagram calculated according to the change of FRET with HC@Z@A/DPH nanocomposites amount is shown in Figure 3D. The kinetic parameters values using HC@Z@A/DPH nanocomposites as substrates were K_m_ (268.01 nM) and k_cat_/K_m_ (3410 mM^−1^ s^−1^). The catalytic efficiency (k_cat_/K_m_) for HC@Z@A/DPH nanocomposites substrate is almost identical to that for the QDP substrate in QDP‐based HNE assay,^[^ ^6^ ^]^ but the binding affinity for HC@Z@A/DPH nanocomposites is slightly weaker than that of the QDP. The so‐called skipping model of activity was responsible for it, in which the enzyme turns over all of the bound peptide substrates on the surface of the nanoparticle before spreading to the neighboring nanoparticle‐substrate conjugate. Compared with QDP,^[^ ^6^ ^]^ the skipping model of activity in HC@Z@A/DPH nanocomposites was confronted with resistance from the track H3 to a certain extent. The HC@Z@A/DPH nanocomposites could serve as substrates for HNE enzymatic study.

To investigate the potential applications of HC@Z@A/DPH nanocomposites in drug discovery, we performed kinetic characterization of sivelestat, a well‐known HNE inhibitor, using HC@Z@A/DPH nanocomposites as a reporter. Sivelestat is the only commercially available selective HNE inhibitor. As shown in Figure 3E; Figure S5B (Supporting Information), sivelestat significantly inhibited HNE and exhibited high inhibitory potency (IC_50_ = 14.77 ± 2.53 nM), which was in agreement with the previously reported QDP‐based HNE assay. Therefore, the HC@Z@A/DPH nanocomposites probe can be utilized in HNE inhibitors screening and drug discovery. In addition, the interaction between HNE and Sivelestat has been studied by molecular dynamics (MD) simulation. The simulation process has been stated in the MD simulation process in the experimental section. By visualizing the trajectory and structure of the complex in VMD,^[^ ^41^ ^]^ it is observed that the Sivelestat and the HNE bind together and their positions relative to each other are steady during the simulation. Hydrogen bonds have been analyzed and twenty hydrogen bonds have been found during the simulation. However, most of them appear very occasionally. Only the hydrogen bond between the amino acid residue GLY193 and the sulfonyl group of Sivelestat as well as the hydrogen bond between the amino acid residue PHE41 and the amino group of Sivelestat exists persistently during the simulation, as presented in Figure 3F. Moreover, the interaction energy between HNE and Sivelestat has been calculated and an average value of −186.964 kJ mol^−1^ has been obtained based on the data from the last 7 ns of the MD simulation. In summary, the MD simulation results reveal the inhibitory effect of Sivelestat on HNE

### 2.5 Performance Assessment of HNE Determination In Vitro

We first optimized the conditions of this 3D DNAzyme motor nanodevice to ensure high detection performance. Among them, an important consideration is whether the pH value environment can ensure the disintegration of HC@Z@A/DPH nanocomposites to liberate sufficient hairpins and PCPs for subsequent EFEA reaction and chemokinetic treatment. The influence of pH on HC@Z@A/DPH dissociation was examined by the developed FRET‐based biosensor. As seen from Figure S6A (Supporting Information), the FRET signal continued to increase as pH value decreased, revealing that more HC@Z@A/DPH are disintegrated in these cases. The FRET signal reaches a stable at pH 5.5, which is equal to the lysosome environment and tumor micro‐environment. Therefore, we finally selected the pH value of Tris‐HCl at 5.5. Additionally, the self‐powered FRET increased gradually as incubation time prolonged. One observed that the accumulated FRET signal reached a plateau after 120 min, indirectly confirming the high amplification of DNA walker cascaded EFEA strategy and fast kinetics of HC@Z@A/DPH (Figure S6B, Supporting Information). Moreover, the ratio of track H3 to DP walking strand affects the efficiency of the HNE identification and DNA walker operating, and the amount of subsequent HCR primer. The ratio of H3 to DP strand was thus studied. As can be seen (Figure S6C, Supporting Information), the FRET signal positively correlates with the ratio of track H3 to DP walking strand, and reached a plateau at 20:1, indicating that DNA walker and EFEA amplification can be essentially completed.

Under suitable conditions, the assay performance of 3D DNAzyme motor nanodevice HC@Z@A/DPH for HNE was studied. As seen in Figure 3G,H, when the HNE target concentration rises, the fluorescence intensity of Cy5 increases gradually but the fluorescence intensity of Cy3 decreases correspondingly. By plotting the F_D_/F_A_ values against HNE concentration, a good linear relationship can be obtained in the range of 1–30 ng mL^−1^. The correlation equation could be expressed as F_D_/F_A _= 0.1958 + 0.0878 C_HNE_ with the correlation coefficient of R^2 ^= 0.996. The detection limit was calculated to be 0.026 pM (S/N = 3), which is much lower or at least comparable to other methods used for HNE detection (Table S3, Supporting Information). The high sensitivity of the 3D DNAzyme motor nanodevice can be attributed to the DNA walker cascaded EFEA self‐powered FRET amplifier, and low nonspecific adsorption and background signals provided by HC@Z@A/DPH. The 3D DNAzyme motor transformed stoichiometrically and amplified the target HNE recognition event into a repeated formation of the long DNA concatamer to generate self‐powered FRET amplified signals.

To examine the selectivity of the 3D DNAzyme motor nanodevice HC@Z@A/DPH nanocomposites, we investigated the specificity of HC@Z@A/DPH nanoprobe by monitoring its fluorescent responses to HNE and other proteases (PR3, CPG, BChE, AChE, pepsin). Figure 3I shows the fluorescence changes for the different types of targets, each with a concentration of 25 ng mL^−1^. It was observed that no significant fluorescence changes were obtained for these control proteases. The FRET intensity induced by HNE was increased by ≈9 times in comparison with that of produced by control proteases. Results between HNE and other protease groups were greatly different, which was statistically significant (*p* < 0.001). This suggests that this 3D DNAzyme motor nanodevice can identify HNE from other proteases. Moreover, the mixture of HNE with other proteases generates FRET signal intensity identical to that for the addition of HNE alone, suggesting the good anti‐interference capability of the 3D DNAzyme motor nanodevice. Additionally, the largest value of coefficients of variation for triplicate measurements was 5.6%, indicating the excellent reproducibility of the 3D DNAzyme motor nanodevice for its application in the detection of target HNE. This result suggests that the 3D DNAzyme motor nanodevice HC@Z@A/DPH could offer satisfactory specificity and reproducibility, and possesses a great potential to be as a ratiometric fluorescent nanoprobe for HNE detection.

### 2.6 Fluorescence Imaging of HNE in Living Cells and in Tumor Model Mice

**Figure 4:**
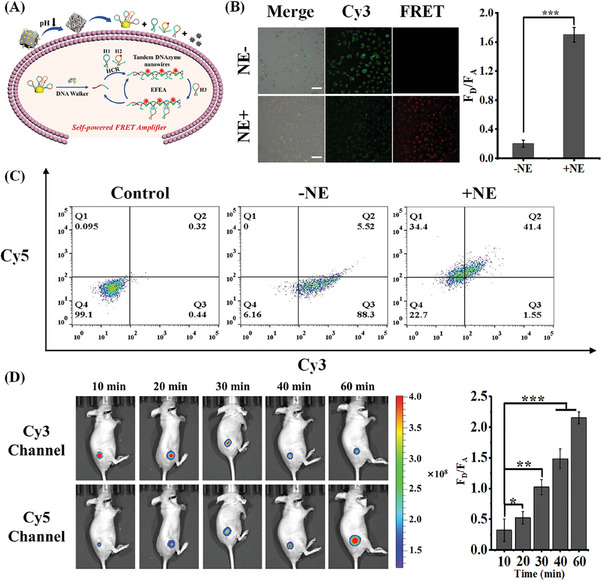
A) Schematic diagram of imaging HNE in living cells. B) Fluorescence image of A549 cells (without or with HNE preincubation) treated with HC@Z@A/DPH nanocomposites. Quantitative determination of fluorescence signal change using the F_D_/F_A_. C) Flow cytometry quantitative analysis of A549 cells (without or with HNE preincubation) treated with HC@Z@A/DPH nanocomposites. D) Fluorescence images taken from A549‐tumor‐bearing nude mice from the Cy3 and Cy5 channel after injection with HC@Z@A/DPH nanocomposites in situ at various time points (10, 20, 30, 40, 60 min). Quantitative determination of fluorescence signal change using the F_D_/F_A_. * *p* < 0.05, ** *p* < 0.01, and *** *p* < 0.001.

For now, few HNE probes can be used for the amplification and imaging of HNE in living cells and in vivo, which limits the application of in situ diagnostics for early tumors. In this regard, HC@Z@A/DPH nanocomposites were exploited for imaging HNE in A549 lung tumor cells (**Figure** **4**A). Before imaging HNE in living cells was studied, the cytotoxic activity of HC@Z@A/DPH nanoprobes against A549 cell lines was assessed, in which it found that there is no obvious cytotoxicity, even at a high concentration of 30 µg mL^−1^ after incubation for 24 h (Figure S8B, Supporting Information). For living cells imaging, A549 cells were first cultured and incubated with 50 nM HNE in 1640 medium (90% RPMI 1640 medium and 10% FBS and 1% penicillin/streptomycin antibiotic solution), followed by washing several times. The cells were then exposed to HC@Z@A/DPH nanocomposites (final concentration 20 µg mL^−1^) in the medium for 2.0 h. As shown in Figure 4B, A549 cells incubated with HC@Z@A/DPH nanocomposites exhibited a strong red fluorescence and a weak green fluorescence. In contrast, a strong green fluorescence appeared, whereas the red fluorescence sharply disappeared when no HNE was added to the medium. Moreover, we quantified the fluorescence values of Cy3 and Cy5 by flow cytometry (Figure 4C). One observed that the Cy5 fluorescence signal increased significantly and the corresponding Cy3 fluorescence became weaker in HNE‐treated cells, suggesting that the self‐powered FRET strategy successfully occurred. By contrast, no significant Cy5 fluorescence appeared in cells that were not treated with HNE. Based on the above results, we subsequently assessed the imaging capability of HC@Z@A/DPH nanoprobes in vivo. As shown in Figure 4D, after HC@Z@A/DPH nanocomposites were injected into the mouse tumor model, the red fluorescence enhanced step by step, whereas the green fluorescence was notably weak in the primary tumor location in a time‐dependent manner. The standpoint is that HNE has the power to promote tumor cell proliferation and invasion through the PDGFR‐PI3K signaling pathway. The in vivo study also suggested that the expression of HNE level was in association with the lung tumor occurrence and progress.

### 2.7 Analysis of HNE Activity in Human Tissue Samples

**Figure 5:**
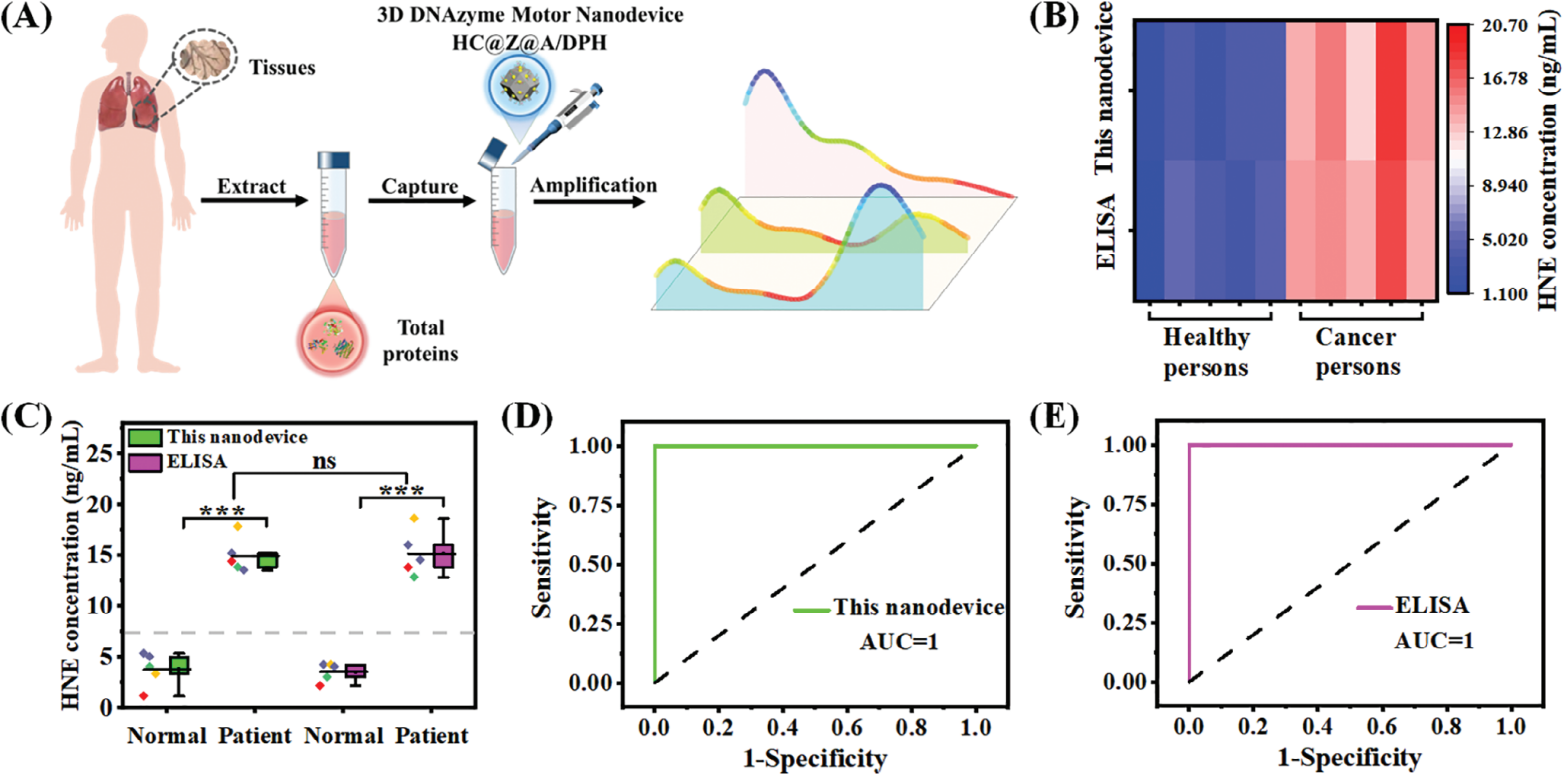
A) Schematic diagram of the proposed 3D DNAzyme motor nanodevice for quantitative assay of HNE activity in lung tissues (5 lung cancer patients and 5 healthy participants). B) The Heatmap of HNE expression from different lung tissues by the proposed nanodevice and ELISA. C) Boxplots of HNE expression distribution in different lung tissues by the proposed nanodevice and ELISA. D, E) ROC analysis to evaluate the accuracy of this assay in discriminating lung cancer patients from healthy individuals by the proposed nanodevice (D) and ELISA (E). ns, no significance, *** *p* < 0.001.

Accurate determination of HNE level is of significance for clinical diagnosis and treatment of pulmonary tumor diseases. The 3D DNAzyme motor nanodevice was conducted to evaluate the clinical applicability of measuring HNE in 5 lung cancer patients and 5 healthy participants (**Figure** **5**A). The respective HNE in each tissue sample was simultaneously measured via both the nanodevice and ELISA (Figure 5B). Notably, the values of HNE measured by the nanodevice were not different from those measured by ELISA for the same tissue sample (Student's t‐test, *p* > 0.05). As seen in Figure 5B, the expression of HNE in lung cancer tissues was significantly higher those in normal control tissues. The HNE concentrations obtained from 3D DNAzyme motor nanodevice showed a strong positive correlation with those obtained from ELISA (Figure S7, Supporting Information). Additionally, the Student's t‐test was used in different tissue groups for statistical analysis (*p* < 0.001). As seen in Figure 5C, HNE levels in patients with lung cancer were upregulated compared with those in healthy controls. The HNE values in lung cancer tissues were on average 3.5 times higher than in normal control tissues, which were in line with those obtained from the ELISA method. Receiver operating characteristic (ROC) analysis has also been performed to determine whether the 3D DNAzyme motor nanodevice yields better accuracy in differentiating HNE levels from lung cancer tissues and normal control tissues. As shown in Figure 5D, the area under the curve (AUC) values for the nanodevice were calculated to be 1, which was in line with those obtained from the ELISA method (Figure 5E, AUC = 1), indicating that the proposed nanodevice has a discriminatory ability to differentiate groups of patients and healthy controls with high accuracy.

### 2.8 CDT Efficacy and Therapeutic Mechanism of HC@Z@A/DPH Nanocomposites

Lung carcinogenesis is a complex process in an unregulated inflammatory environment. Neutrophil elastase (NE), an important regulator of inflammatory processes, directly triggered tumor cell proliferation in human lung adenocarcinoma A549 cells. The PCP nanodots in HC@Z@A/DPH nanocomposites cause apoptosis through regulation of protein expression related to apoptosis by ROS‐mediated pathway. To assess the killing effect and chemodynamic efficiency of HC@Z@A/DPH nanocomposites on lung cancer cell A549, live/dead (calcein AM and propidium iodide) staining, and MTT assay were performed to assess the cellular viability and proliferation. Fluorescence imaging of cells revealed that HC@Z@A/DPH nanocomposites elicited cancer cell death (Figure S8A, Supporting Information). MTT assay showed that cell viability decreased sharply to ≈20% after a 24 h incubation exposure period with 200 µg/mL HC@Z@A/DPH nanocomposites, relative to the controls (Figure S8B, Supporting Information). Whereas, HC@Z@A/DPH nanocomposites displayed less toxicity against normal cells (Figure S8C, Supporting Information). The results revealed that HC@Z@A/DPH nanocomposites can serve as a powerful chemodynamic therapy nanoagent, and possess excellent antitumor potency.

**Figure 6:**
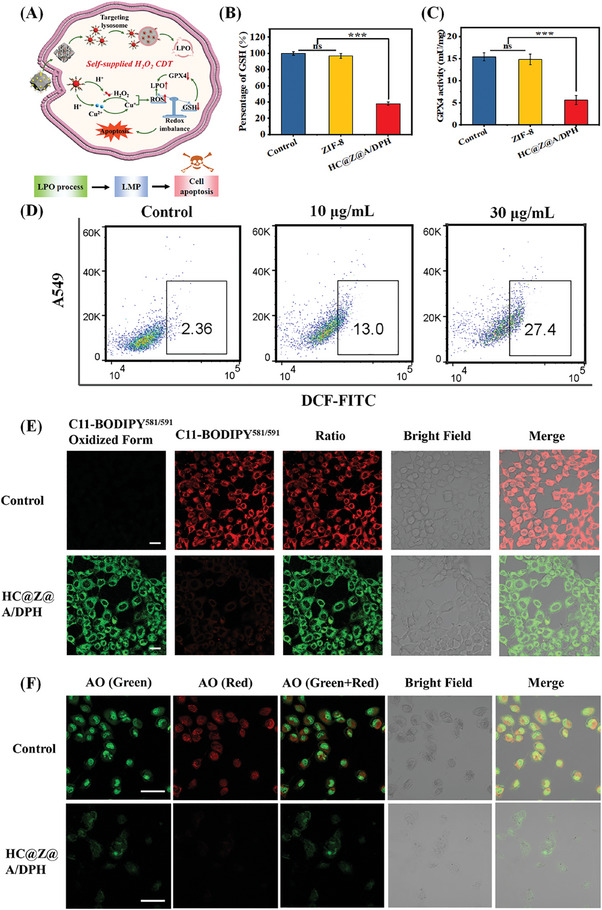
A) Schematic mechanism of HC@Z@A/DPH‐enabled A549 cancer cell ferroptosis. B) GSH level. C) GPX4 activity. and D) Intracellular ROS measurement by flow cytometry. E) CLSM images of C11‐BODIPY^581/591^, the scale bar is 20 µm. F) CLSM images of AO staining, The scale bar represents 50 µm. ns, no significance, ***p < 0.001.

After endocytosis into tumor cells, the HC@Z@A/DPH nanocomposites gradually disintegrated in the weakly acidic tumor micro‐environment and released the PCP nanodots as an activatable agent for enhanced CDT by self‐supplying H_2_O_2_. The PCPs could target lysosomes and decompose under acidic conditions, producing Cu^2+^ and H_2_O_2_. The Fenton‐like reaction between Cu^2+^ and H_2_O_2_ generates ROS (•OH) and Cu^+^, and that between Cu^+^ and H_2_O_2_ generates ROS and Cu^2+^, forming a self‐supplying cyclic catalysis effect (**Figure** **6**A). Cu^2+^ reacts with glutathione (GSH) to generate Cu^+^ and GSSG. Simultaneously, the GSH consumption decreases the glutathione peroxidase 4 (GPX4) expression (Figure 6B,C). The generated ROS (•OH) induced lysosomal lipid peroxidation (LPO) of the lysosomal membrane, resulting in lysosomal membrane permeabilization (LMP) and thus causing cell apoptosis through a lysosome‐associated pathway (Figure 6D,E). Studies have shown that lipid peroxidation affects immune cell function to modulate tumor immunity and antitumor ability. The HC@Z@A/DPH nanocomposites‐enticed lipid peroxidation was evidenced by C11‐BODIPY^581/591^, a lipophilic and good penetration into lipid bilayer ratiometric fluorescent probe. It can turn red fluorescence to green when the product lipid hydroperoxide (LHP) is present. As shown in Figure 6E, the red fluorescence decreased and the green fluorescence significantly increased after A549 cells were incubated with HC@Z@A/DPH nanocomposites and C11‐BODIPY^581/591^ dye. The result successfully demonstrated that the HC@Z@A/DPH nanocomposites is able to bring about lysosomal lipid peroxidation (LPO) of the lysosomal membrane.

To determine whether PCPs could target lysosomes, the path of the PCPs was tracked. Here, fluorescence‐labeled PCPs (0.5 µg mL^−1^) were employed to check the intracellular distribution after incubation with A549 cells, there is no obvious cytotoxicity at 0.5 µg mL^−1^ after incubation for 48 h. The green fluorescence was from the PCPs, and the red fluorescence was from the stained lysosomes (Figure S9, Supporting Information). It was observed that the green fluorescence mostly coincided with the red fluorescence, suggesting the PCPs could target lysosomes. With an increase in the incubation time, the fluorescence coincident range is almost constant, indicating that the PCPs do not occur when the lysosome escapes. Whereafter, the acridine orange (AO) staining method was employed to assess the integrity of the lysosomal membrane when A549 cells were incubated with HC@Z@A/DPH nanocomposites. After AO staining, cytoplasm and nuclei emit green fluorescence, and intact lysosomes emit red fluorescence. As shown in Figure 6G, the untreated control cells displayed both red and green fluorescence. Inversely the treatment of A549 cells with HC@Z@A/DPH nanocomposites displayed weak or insignificant red fluorescence. The phenomenon confirms the damage of lysosomes, which was imputed to the •OH generation by HC@Z@A/DPH nanocomposites within endo/lysosomes and the consequent lysosomal LPO. Lysosomal membrane permeabilization (LMP) caused by the damage of membrane integrity could permit the released cathepsins to enter into cytosol and thus generate cancer cell apoptosis. The lysosomal rupture caused by •OH‐induced LPO is one of the important mechanisms in charge of HC@Z@A/DPH nanocomposites‐mediated tumor cell killing.

### 2.9 In Vivo Antitumor Studies

**Figure 7:**
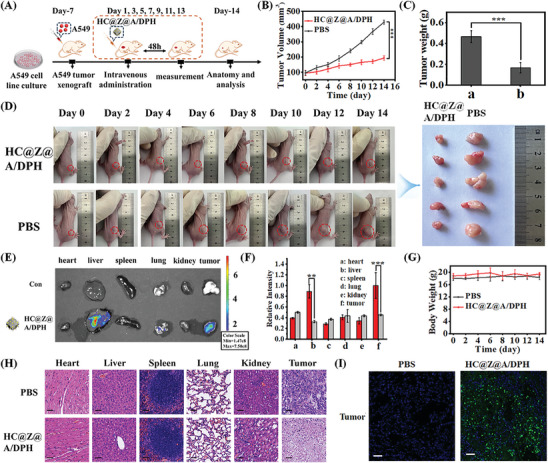
A) Schematic representation of anti‐tumor manipulation in nude mice within 14 days of the HC@Z@A/DPH nanocomposites. B) Volume plot of tumor changes in mice over 14 days in the HC@Z@A/DPH and PBS group. C) Plot of the mass difference of mouse tumors after 14 days in PBS (a) and HC@Z@A/DPH (b) groups. D) Photographs of tumor changes in nude mice within 14 days and tumors in nude mice after 14 days. ***p < 0.001 versus the control group. E) Fluorescence image of the tumor and various tissues dissected from control or HC@Z@A/DPH‐treated model mice 2 h after In vivo imaging: a. heart b. liver c. spleen d. lung e. kidney. f. tumor. F) Quantification of the relative fluorescence intensity in the tumor and each organ. G) Plots of body weight changes over the 14 days in the HC@Z@A/DPH and PBS groups. H) H&E‐stained images of various tissues and tumor slices obtained from different groups of mice. I) Fluorescence images of TUNEL‐stained tumor slices after HC@Z@A/DPH‐treated. Cell nuclei were stained with DAPI (blue fluorescence). Green fluorescence indicates TUNEL‐positive cells. The scale bar represents 50 µm. ** *p* < 0.01 and *** *p* < 0.001.

Having demonstrated its in vivo targeting behavior, we explored the CDT efficacy of the HC@Z@A/DPH nanotheragnostic agent. Whether the HC@Z@A/DPH nanocomposites could efficiently inhibit tumor growth was evaluated in a mouse model after intravenous administration (i.v.). The intravenous administration was performed every other day for 14 days. As shown in **Figure** **7**A, the tumor growth rate of mice injected with PBS was rapid, while the tumor growth rate of mice injected with HC@Z@A/DPH nanocomposites was significantly suppressed (Figure 7B,D). Subsequently, the tumor tissues were isolated from the tumor‐bearing mice after final administration, and significant inhibition in tumor sizes and weights was observed with HC@Z@A/DPH treatment (Figure 7C,D). The significant inhibitory effect of the HC@Z@A/DPH nanocomposites on the growth of tumors was mainly attributed to the effective tumor accumulation and the highly toxic ROS (•OH) generation. The HC@Z@A/DPH nanocomposites effectively accumulate in most of the cancer tissues mainly through the enhanced permeation and retention effect (EPR).

In tumor‐bearing mice models, the biodistribution of HC@Z@A/DPH nanocomposites was investigated. In vivo results revealed that besides the accumulation in the liver, the tumor uptake of HC@Z@A/DPH nanocomposites was significantly (*p* ≤ 0.001) higher than irrelevant tissue and organ at 24 h post i.v. injection (Figure 7E,F). The liver is an important apparatus of the body that has the function of detox. It plays an important role in the metabolism of substances in the body. The high tumor accumulation of HC@Z@A/DPH nanocomposites provides an enhanced CDT application in vivo. In addition, no significant change in body weight was found among mice receiving i.v. injection of HC@Z@A/DPH nanocomposites therapy (Figure 7G). Hematology and biochemical analysis exhibited no apparent toxicity of the HC@Z@A/DPH nanocomposites in mice at the tested period (Figure S10, Supporting Information). Moreover, no visible histological damage was observed in the major organs of HC@Z@A/DPH nanocomposites‐treated mice (Figure 7H). Hematoxylin and eosin (H&E) staining was performed to assess the therapeutic effect, in vivo antitumor studies revealed that HC@Z@A/DPH nanocomposites could result in desirable CDT effects and destroy primary tumors. Moreover, a western blot was employed to examine protein expression within tumor tissues. Observed from Figure S11 (Supporting Information), the HC@Z@A/DPH nanocomposites indeed down‐regulated the HNE level, and caused apoptosis in tumor tissue. Subsequently, HC@Z@A/DPH nanocomposites‐induced apoptosis in tumor tissue was measured by terminal deoxynucleotidyl transferase‐mediated dUTP nick‐end labeling (TUNEL) staining (Figure S10I, Supporting Information). TUNEL staining significantly revealed higher apoptosis rates in HC@Z@A/DPH nanocomposites‐treated animals compared to saline‐treated mice. In consequence, the results certify the feasibility of HC@Z@A/DPH nanocomposites as chemodynamic therapy agents with enhanced antitumor activity for cancer therapy.

## 3 Conclusion

In summary, we have demonstrated the peptide‐assembled 3D DNAzyme motor nanodevice HC@Z@A/DPH for self‐powered FRET amplified profiling of HNE and self‐supplied H_2_O_2_ enhanced chemodynamic therapy in lung cancer diagnosis and treatment. The developed 3D DNAzyme motor nanodevice has significant advantages: 1) The target HNE catalyzes the cleavage of DNAzyme‐peptide detection probe to convert the enzyme signal to the DNA signal, which can be highly amplified by DNA walker cascaded EFEA self‐powered amplification strategy and detected in a FRET manner featuring excellent accuracy and convenience. 2) FRET ratiometric output is employed to avoid false positive signals from complex samples, the 3D DNAzyme motor‐based self‐powered FRET concatamer endows this assay with a high sensitivity and accuracy for imaging of HNE in cell and mice. 3) The 3D DNAzyme motor nanodevice can be used for the kinetic study and inhibition assay, and it can also differentiate the HNE levels in the tissues of healthy persons and lung cancer patients, with promising applications in HNE‐related cancer diagnosis and therapeutics. 4) It may become a universal platform for the detection of other enzymes by designing appropriate DNAzyme‐peptide substrates. 5) The PCPs enhanced CDT through a Fenton‐like reaction generating ROS by self‐supplying H_2_O_2_, which increased local oxidative stress and effectively consumed the GSH in tumor cells, synergically resulting in the accumulation of LPO and cell apoptosis. 6) The HC@Z@A/DPH increased PCPs intracellular delivery. The PCP could target the lysosome of tumor cells through lysosomal targeted peptides, reducing lysosome escape, enabling high tumor accumulation and inhibiting tumor growth, and down‐regulating the HNE expression with high CDT. In summary, this developed 3D DNAzyme motor nanodevice was reliable and had a great potential application in clinical diagnosis and therapy of HNE‐related lung tumors, and drug discovery.

## 4 Experimental Section

### Materials

Oligonucleotides (Table S1, Supporting Information) and streptavidin were obtained from Sangon Biotechnology Co., Ltd. Biotinylated polypeptide (Bio‐APEEIMAQKC‐CONH_2_) was purchased from Sinopeptide Biochemical Co., Ltd. (Zhejiang, China). 2‐methylimidazole and zinc acetate were obtained from Aladdin (Shanghai, China). Copper(II) chloride (CuCl_2_•2H_2_O), FeSO_4_•7H_2_O, NaBH_4_, sodium hydroxide, and polyvinylpyrrolidone (PVP), sodium chloride and methanol were obtained from Sinopharm Chemical Reagent. Tri‐(2‐carboxyl ethyl) phosphine Hydrochloride (TCEP) and hydrogen tetrachloroaurate (III) trihydrate (HAuCl_4_·3H_2_O, 99% purity) were purchased from Sigma‐Aldrich. Human neutrophil elastase (HNE) was purchased from Qiyi Biotechnology Co., Ltd. (Shanghai, China). Fetal bovine serum was purchased from Jinan Saiaomei Biotechnology Co., Ltd. (China). Penicillin/streptomycin antibiotic solution, RPMI 1640 medium, 30% hydrogen peroxide, Tween‐20, BSA, and proteinase 3(PR3), cathepsin G (CPG), butyrylcholinesterase (BChE), acetylcholinesterase (AChE), trypsin, and pepsin were obtained from Sangon Biotechnology Co., Ltd. 2‐mercaptoethanol was purchased from Yingsi (Beijing) Medical Device Co., Ltd. (China). All Balb/c nude was 4 weeks old and purchased from Beijing Vitonglihua Experimental Animal Technology Co., Ltd. (China). All solutions were prepared and diluted with ultra‐pure water (≥18.25 MΩ cm).

### Instruments

Ultraviolet‐visible (UV–Vis) absorption spectra were achieved with a UV‐2550 spectrophotometer (Shimadzu, Japan). The morphology was observed by a JEM‐2100F transmission electron microscope (TEM), and scanning electron microscope (SEM) images were recorded on a scanning electron microscope (Thermo Fisher Scientific FIB‐SEM GX4), and the microscope was equipped with an energy dispersive spectrometer (EDS). Energy dispersive X‐ray photoelectron spectroscopy (XPS) was carried out on an ESCALAB MK II X‐ray photoelectron spectrometer. The fluorescence spectrum was acquired through an FLS1000 fluorescence spectrometer (Edinburgh Instruments). Gel electrophoresis images were performed on the ChemiDoc XRS+ imager (Bio‐RAD, USA). FT‐IR spectra were measured by Nicolet‐5700 infrared spectrometer (Shanghai, China). Confocal fluorescence image experiments were carried out on an LSM 880 confocal laser scanning fluorescence microscope (Carl Zeiss, Germany). The fluorescence of the cell samples was measured using a Guava easyCyte 6‐2L flow cytometer (Millipore, USA). The cell viability measurements were performed on an ELx808 (BioTek, U.S.A.) photometer. In vivo, fluorescence images were acquired via the IVIS SPECTRUM imaging system.

### Preparation of Peptide‐Modified Copper Peroxide (PCP) Nanoparticles

The copper peroxide (CP) nanoparticles were first synthesized as follows: 2.0 g PVP was added to 20 mL of freshly prepared copper chloride (0.01 m) under continuous stirring. After that, 20 mL of NaOH solution (0.02 m) and 450 µL hydrogen peroxide (30 wt.%) were added in drops under gentle stirring followed by incubation for 0.5 h. Finally, the as‐prepared copper peroxide (CP) nanoparticles were concentrated by a superfilter, and collected by freeze‐drying.

2 mL of CPs was mixed with 2 mL of lysosomal targeting peptide (SH‐FFRIKFERQ) aqueous solution (1.25 mg mL^−1^) under vortex agitation, and incubated at room temperature for 10 h. The peptide‐modified CPs (PCPs) were purified and concentrated using a superfilter tube (MWCO:100 kDa) to cut off the free peptide. Finally, the PCPs were redispersed in PBS (10 mM, pH 7.4) and stored at 4 °C for future use. The concentration of PCPs was determined by ICP‐MS analysis.

### Synthesis of Hairpins&PCPs@ZIF‐8@Au (HC@Z@A)

1.0 µM hairpin probes (H1, H2, H3) were first mixed to the PCPs suspended in PBS buffer (10 mM, pH 7.4). Then, 2 mL zinc acetate solution (50 mM) and 2‐methylimidazole (0.5 M) were added under gentle stirring followed by incubation for 5 h at room temperature. Subsequently, the resulting hairpins&PCPs@ZIF‐8 (HC@Z) were centrifuged at 8000 rpm, and resuspended in PBS solution (10 mM, pH 7.4) for future use.

150 µL of HAuCl_4_·3H_2_O (5 mM) was mixed with 5 mL of HC@Z at room temperature for 40 min. After that, 1 mL of 3.6 mg NaBH_4_ was added to reduce the precursor under stirring. After 2 h, the product was centrifuged and washed three times with fresh methanol. Finally, the hairpins&PCPs@ZIF‐8@Au (HC@Z@A) were obtained by vacuum drying at 80 °C for 10 h.

### Synthesis of Hairpins&PCPs@ZIF‐8@Au/DP&H3 (HC@Z@A/DPH) Nanocomposites (also termed 3D DNAzyme Motor Nanodevice)

To prepare the hairpins&PCPs@ZIF‐8@Au/DP&H3 (HC@Z@A/DPH) nanocomposites, the HC@Z@A nanoparticles were first re‐suspended in 0.15 M PBS solution. Then 25 µL of biotinylated polypeptides (1 µM) were added to the HC@Z@A solution (100 µL, 20 µg mL^−1^). Whereafter, the solution was incubated on a rocking shaker for 3 h to allow the poly‐peptide to bind to the AuNP surface of HC@Z@A nanoparticles through an Au‐thiol (at cysteine) interaction. After the washing steps (5 min at 10,000 rpm, resuspension in PBS, 0.05% Tween 20, 0.1% BSA), it was incubated on a shaker with thiol‐modified DNAzyme walker strand (5′‐SH, 3′‐biotin, 25 µL, 1 µM) for 1 h. After the washing steps (5 min at 10 000 rpm, resuspension in PBS, 0.05% Tween 20, 0.1% BSA), streptavidin (1 mg mL^−1^) was added in the solution of bifunctional HC@Z@A at 37 °C for 1 h. After the streptavidin‐biotin reaction on the AuNP surface of HC@Z@A nanoparticles, the DNAzyme‐peptide conjugates were prepared on the AuNP surface of HC@Z@A nanoparticles to form HC@Z@A/DP, meanwhile, residual streptavidin was removed by the centrifugal methods (5 min at 10 000 rpm) with 3 times repetition. Next, the prepared HC@Z@A/DP was redispersed in PBS (10 mM, pH 7.4). After that, 50 µL of 10 µM thiolated H3 (track substrate) was added to the HC@Z@A/DP solution and vibrated at 37 °C for 5 h. The superfluous H3 was removed by centrifugation (5 min at 10 000 rpm) with 3 times repetition. Furthermore, for the passivation of the Au surface, 3% BSA in PBS was added overnight at 4 °C. After the washing steps (5 min at 10 000 rpm, resuspension in PBS, 0.05% Tween 20, 0.1% BSA), HC@Z@A co‐loaded with the DNAzyme‐peptide conjugates and H3. The obtained hairpins&PCPs@ZIF‐8@Au/DP&H3 nanocomposites, which were shortly named as HC@Z@A/DPH, were termed 3D DNAzyme motor nanodevices.

### HNE In Vitro Testing

The prepared HC@Z@A/DPH nanocomposites (termed 3D DNAzyme motor nanodevice) were dispersed into 15 mM Tris‐HCl buffer solutions (pH 5.5) at a concentration of 500 µg mL^−1^. Then various‐amount HNE was added into 50 µL of HC@Z@A/DPH (30 µg mL^−1^) solution and incubated for 2 h at 37 °C. Subsequently, the reaction solution was introduced to fluorescence spectroscopy to detect the HNE activity. The emission spectra were collected from 550 and 700 nm with excitation wavelengths of 540 nm. The maximum fluorescence emission was at 567 nm (Cy3) and 667 nm (Cy5), respectively. Moreover, to investigate its specificity, we used other enzymes to replace HNE, the fluorescent intensity was recorded using the aforementioned procedure.

### Gel Electrophoresis Analysis and Western Blotting

The DNA walker cascaded EFEA amplification was investigated by 12% natural polyacrylamide gel electrophoresis. After the reaction, 10 µL of each sample was mixed with 2 µL of 6× loading buffer. Then 12 µL of the mixture was transferred into the native polyacrylamide gel. Subsequently, electrophoresis was performed in 5× TAE buffer using gel red as the stain at 200 V for 40 min. Finally, the gel electrophoresis was transferred to the Gel Doc XR+ System for imaging.

The total protein concentrations were calibrated with BCA protein assay kit (Beyotime, P0009). The total proteins from tumor tissue were separated by sodium dodecyl sulfate‐polyacrylamide gel electrophoresis (SDS‐PAGE) and then transferred to a polyvinylidene difluoride (PVDF) membrane (Beyotime, FFP32). The transfer time of these proteins to the PVDF membrane lasted 1 h. In this study, the anti‐NE antibody was E9C9L (1:1000, Cell Signaling Technology, 89 241), and the antibody against β‐actin (1:1000, Signaling Technology, 8457) was used for the internal control. An enhanced chemiluminescence kit (Beyotime, P0018S) was applied according to the manufacturer's instructions, and the membrane was imaged by the ChemiDoc XRS+ imager (Bio‐RAD, USA).

### Kinetic Analysis In Vitro

HNE activity was determined by fluorescence assay as follows: a certain amount of HNE was mixed with 50 µL of the HC@Z@A/DPH solution with different concentrations followed by incubating at 37 °C. The time‐dependent fluorescence spectra were collected from 550 and 700 nm with excitation wavelengths of 540 nm, and the enzymatic hydrolysis of HC@Z@A/DPH nanocomposites was investigated.

### Cell Culture and Imaging

A549 cells were cultured in 1640 medium containing 10% fetal bovine serum and 1% of 10 000 unit^−1^ penicillin/streptomycin antibiotic solution at 37 °C with a humidified atmosphere (5% CO_2_). To investigate the feasibility of intracellular HNE imaging, A549 cells were placed on 15 mm culture dishes. After being washed with sterile PBS, HC@Z@A/DPH probe was added to the A549 cells that were processed with and without HNE respectively, and incubated for 2 h at 37 °C. After cells being washed three times with sterile PBS, fluorescence imaging was performed.

### Cytotoxicity

For the MTT assay, A549 cells were first seeded on 96‐well plates and cultured until they reached the logarithmic growth stage. Then, the medium was discarded, HC@Z@A/DPH probe at different concentrations (30, 50, 100, 150, 200, 250, 300, and 350 µg mL^−1^) were added to 96‐well plates and incubated for 24 h, respectively. Subsequently, 5 mg mL^−1^ 3‐(4,5‐dimathylthiazol‐2‐yl)−2,5‐diphenyltetrazolium bromide (MTT) was added, and the mixture was incubated at 37 °C for 4 h. Finally, 50 µL dimethyl sulfoxide was added, and the absorbance was measured by a microplate reader at 490 nm.

### In Vivo Imaging and Antitumor Analyses

The BALB/c‐nu mice were obtained from Beijing Vital River Laboratory Animal Technology Co., Ltd. All animal studies in our research were approved by the Institutional Ethical Committee of Animal Experimentation, Liaocheng University (Approval number: No. AP2024061401). After 5 weeks of age, the mice were randomly divided into 2 groups of 5–6 each. A549 lung cancer cells with a concentration of 1 × 10^6^ were injected into the thigh of mice to construct a subcutaneous tumor model of mice. Follow‐up experiments were conducted after the tumor size reached 100 mm^3^. Each group received an equal volume of saline or HC@Z@A/DPH probe (100 µg mL^−1^) respectively, and in vivo fluorescence imaging was performed. The animals were evaluated for physical, biochemical, and hematological changes during the 14‐day study. All the experiments on mice were done legally.

### Chemokinetic Therapy

For the effect of CDT on tumors in vivo, we selected A549 tumor‐bearing mice with a tumor size of 100 mm^3^ as the model. The mice were divided into 2 groups with 5 mice in each group: normal saline (control group) and HC@Z@A/DPH probe (treatment group). Mice in the control group were intravenously injected with 200 µL normal saline, and mice in the treatment group were intravenously injected with 200 µL 100 µg mL^−1^ HC@Z@A/DPH. After CDT, one mouse in each group was sacrificed, and histopathological analysis was performed by H&E and TUNEL staining. Tumor volume and body weight were recorded for 14 days after treatment. The tumor volume was calculated using D × d^2^/2, where D and d were the maximum and minimum diameters of the tumor, respectively.

### Measurement of Tissue Samples

Tissue samples from healthy people and lung cancer patients were taken from Liaocheng Third People's Hospital. Total protein was extracted from tissue samples using the nuclear extract kit. Tissue samples were first disrupted and homogenized in a lysis buffer. Then the mixture was incubated for 30 min on ice. Subsequently, the mixture was centrifuged for 10 min at 12 000 rpm to obtain a lysate supernatant containing the total protein extracts. The resultant extracts were aliquoted and stored at −80 °C until measurement. The tissue samples (healthy control or lung cancer patients) experiments were conducted in the same way as the HNE activity detection procedure. The collection of all samples was approved by the Ethical Committee of the Third People's Hospital of Liaocheng.

### Molecular Dynamics (MD) Simulation

The structure of human neutrophil elastase (HNE) (PDB ID: 3Q76) was downloaded from the RCSB protein data bank (https://www.rcsb.org/structure/3Q76). Then the co‐crystallized water and other small molecules were removed to create the initial structure of HNE. The initial structure of Sivelestat was downloaded from the PubChem Compound home (https://pubchem.ncbi.nlm.nih.gov/compound/107706). The online molecular docking tool provided by the Mcule.com platform was used to generate the structures of the HNE and Sivelestat complexes, then the one with the best score was selected as the initial structure of the complex in this work. Subsequently, a cubic water box periodic in XYZ direction was built and the complex was placed at the center. Eleven chloride ions were added to keep the neutrality of the system.

The MD simulation was conducted using the 2021.6 version of GROMACS (Source code archive: https://zenodo.org/records/6801842)^[^ ^42^, ^43^ ^]^ The AMBER99SB‐ILDN force field^[^ ^44^ ^]^ was employed for HNE and chloride ions, and the general AMBER force field (GAFF)^[^ ^45^ ^]^ was used for Sivelestat. The topology file of Sivelestat was created by executing the acpype.py script^[^ ^46^ ^]^ to employ Antechamber module^[^ ^47^ ^]^ of AmberTools 18 and then convert to the GROMACS format. The TIP3P^[^ ^48^ ^]^ water model was used to compute the properties of H_2_O. First, the system went through an energy minimization step with the conjugate gradient algorithm and a tolerance of 100.0 kJ mol^−1^ nm^−1^. Subsequently, a 100 ps MD step was conducted with position restraints imposed on the complex. Finally, an MD step of 20 ns was run for the system, during which the pressure was controlled by the isotropic Parrinello‐Rahman barostat with a time constant of 2.0 ps and the temperature was controlled by the velocity‐rescale method with a time constant of 0.2 ps, respectively. Then, the system went through a 100 ps MD process which was implemented with position restraints imposed on the complex. And the box size was observed, which remained ≈8.4×8.4×8.4 nm^3^ during the step. For the position‐restrained MD and the 20 ns MD runs, the time steps for integration were 1 and 2 fs, respectively, the temperatures were set at 298.15 K, and other parameter settings can be referred to in a previous work.^[^ ^49^ ^]^

## Conflict of Interest

The authors declare no competing financial interest.

## Supporting information

Supporting Information

## Data Availability

The data that support the findings of this study are available in the supplementary material of this article.
