# Supplementary material for: 3D DNAzyme Motor Nanodevice With Self‐Powered FRET Amplifier and Self‐Supplied H2O2 for Enhancing Human Neutrophil Elastase Profiling and Chemodynamic Therapy in Lung Tumor
Source: Adv Sci (Weinh). 2024 Sep 30;11(44):2406599. doi: 10.1002/advs.202406599 (PMC11600284; doi:10.1002/advs.202406599)
Supplement: Supplementary file 1 — Supporting Information [file ADVS-11-2406599-s001.docx]

# 3D DNAzyme Motor Nanodevice with Self-powered FRET Amplifier and Self-supplied H_2_O_2_ for Enhancing Human Neutrophil Elastase Profiling and Chemodynamic Therapy in Lung Tumor

*Huiyan Du, Ensheng Xu, Yihan Xu, Qingwang Xue*, Hongxia Xu*, and Jibin Song**

H.Y. Du, E. S. Xu, Y. H. Xu, X.Q. Xue

Department of Chemistry, Liaocheng University, Liaocheng, 252059, Shandong, China

E-mail: [xueqingwang1983@163.com](mailto:xueqingwang1983@163.com)

1. B. Song

State Key Laboratory of Chemical Resource Engineering, College of Chemistry, Beijing University of Chemical Technology, Beijing 10010, PR China

E-mail: [chem64@163.com](mailto:Chem64@163.com)

1. X. Xu

Department of Clinical Laboratory, The Third People’s Hospital of Liaocheng, Liaocheng, 252059, Shandong, China

E-mail: [xuhx1981@126.com](mailto:wangpin1023@126.com;)


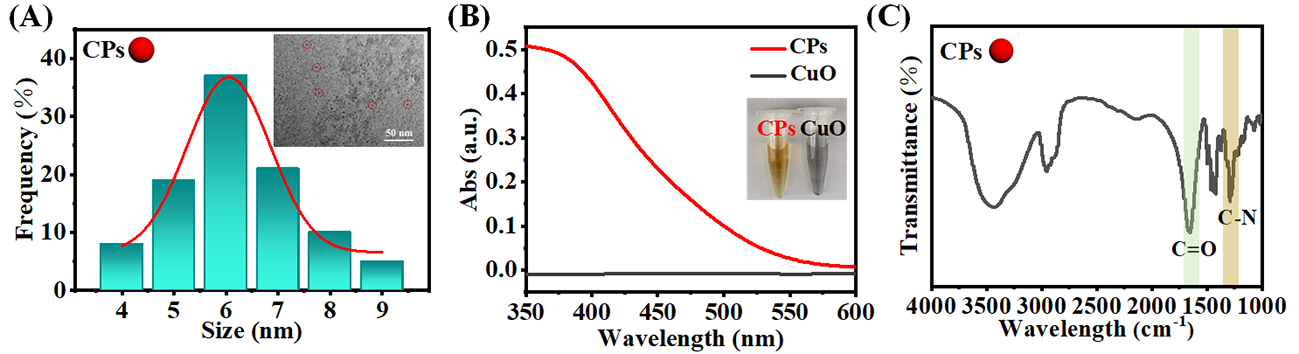


**Figure S1**. **(A)** Particle size of CPs. **(B)** UV-vis absorption of CPs and CuO NPs. **(C)** FT-IR spectra of CPs.


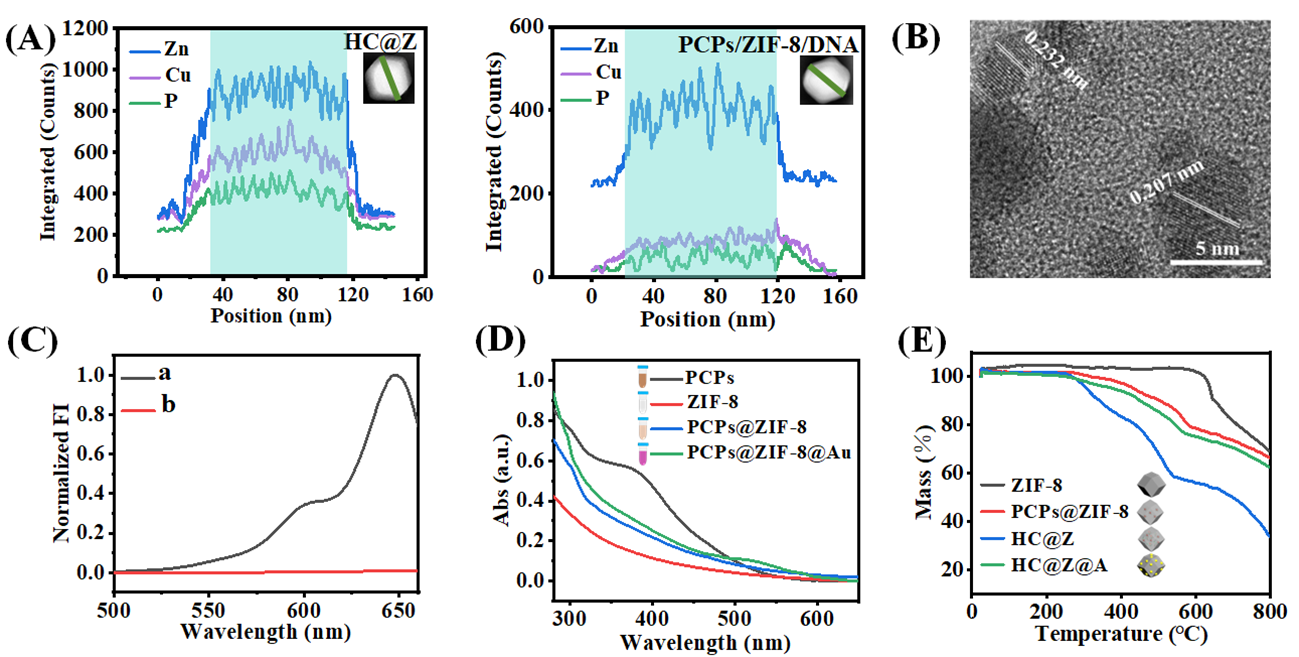


**Figure S2**. **(A)** HAADF and EDS elemental line scanning spectra of Zn, Cu, P elements in hairpins&PCPs@ZIF-8 (HC@Z) and PCPs/ZIF-8/DNA. **(B)** HRTEM of HC@Z@A. **(C)** Normalized fluorescence spectra of the hairpins with Cy5 label (a), and the supernatant after HC@Z@A/DPH nanocomposites preparation (b). **(D)**UV-vis absorption spectrum of PCPs, ZIF-8, PCPs@ZIF-8, PCPs@ZIF-8@Au. **(E)** Thermogravimetric analysis (TGA) curves of ZIF-8, PCPs@ZIF-8 (C@Z), HC@Z and HC@Z@A.


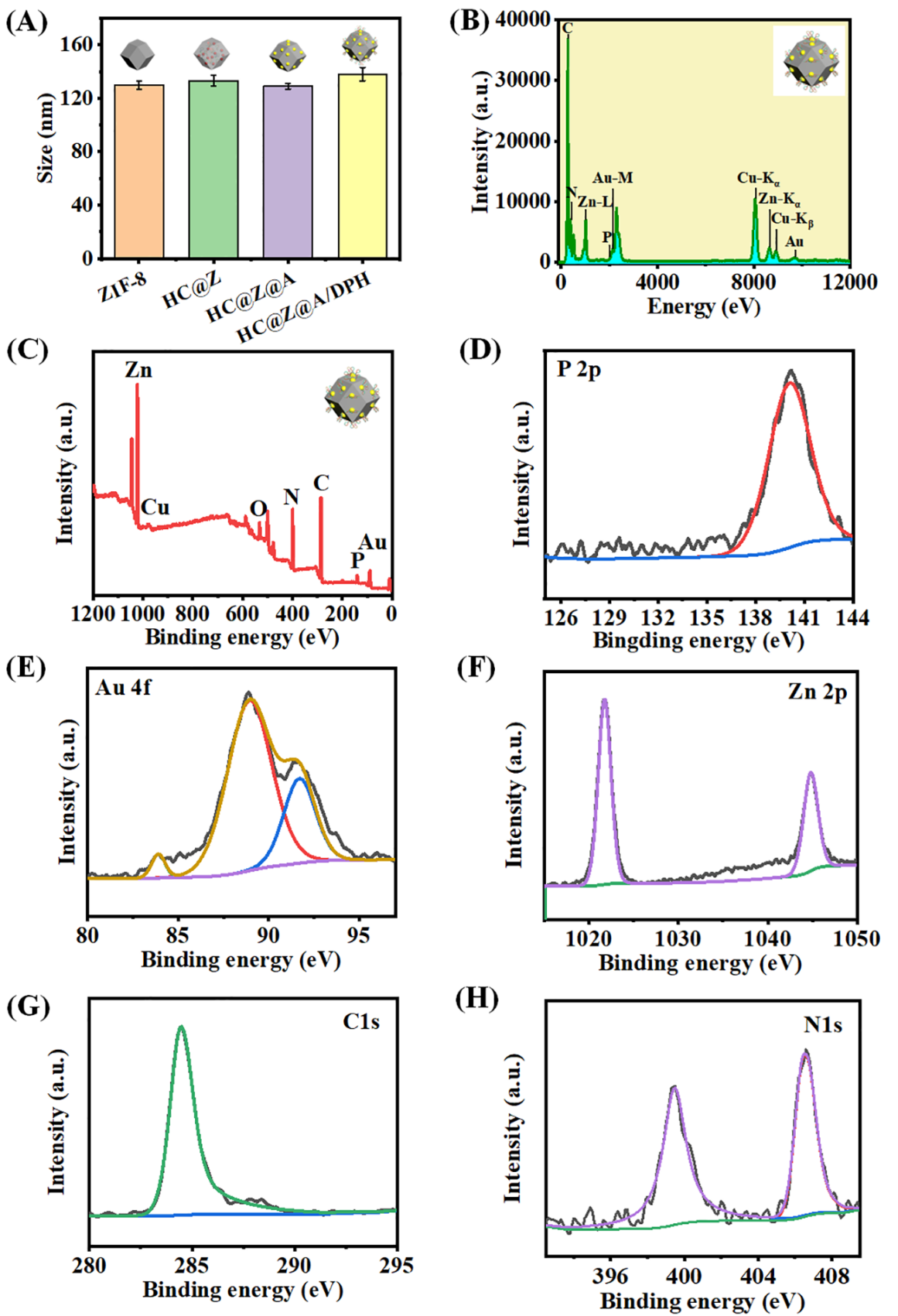


**Figure S3**. **(A)** Diameters of ZIF-8, haipins/PCPs@ZIF-8 (HC@Z), haipins/PCPs@ZIF-8@Au (HC@Z@A), and HC@Z@A/DPH probe. **(B)** Energy-dispersive spectrum of HC@Z@A/DPH probe. **(C)** XPS of HC@Z@A/DPH probe. **(D)** P 2p of the HC@Z@A/DPH. **(E)** Au 4f of the HC@Z@A/DPH. **(F)** Zn 2p of the HC@Z@A/DPH. **(G)** C 1s of the HC@Z@A/DPH. **(H)** N 1s of the HC@Z@A/DPH.


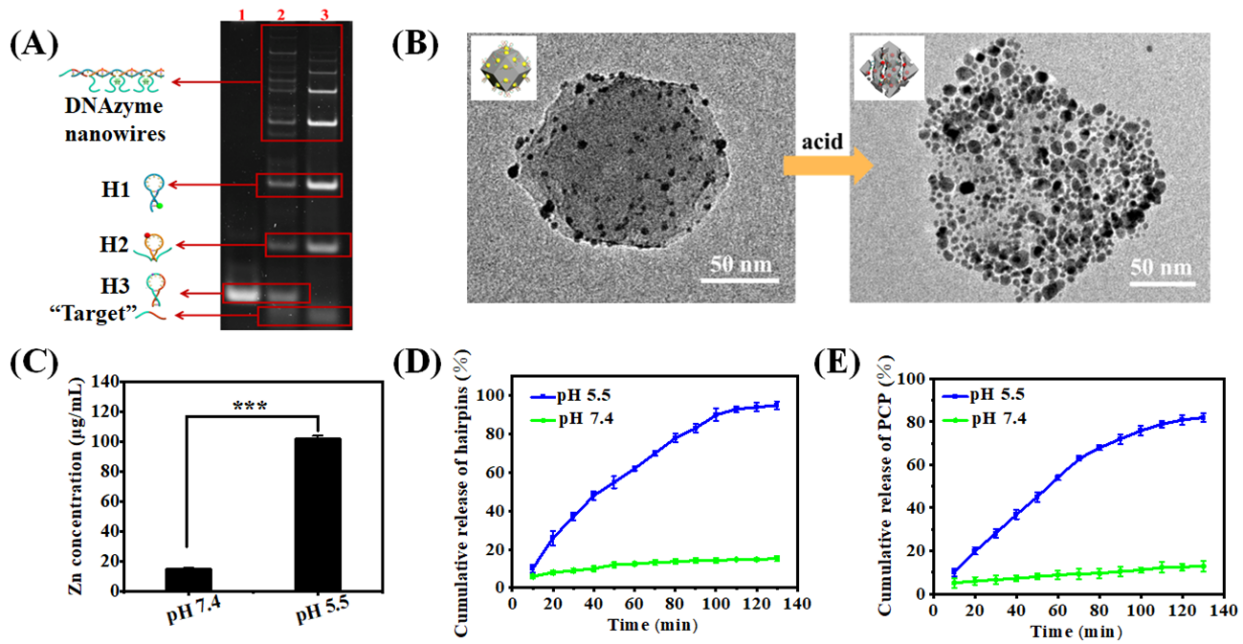


**Figure S4**. (A) The HCR-mediated DNAzyme cleavage and EFEA reaction. **(B)** TEM images of HC@Z@A/DPH nanocomposites after incubation in acid solution. **(C)** Inductively coupled plasma mass spectrometry (ICP-MS) analysis of released Zn^2+^ at pH 7.4 and 5.5. The time-dependent release of **(D)** hairpins and **(E)** PCPs from HC@Z@A/DPH nanocomposites under different pH values. *******p < 0.001 versus the control group.


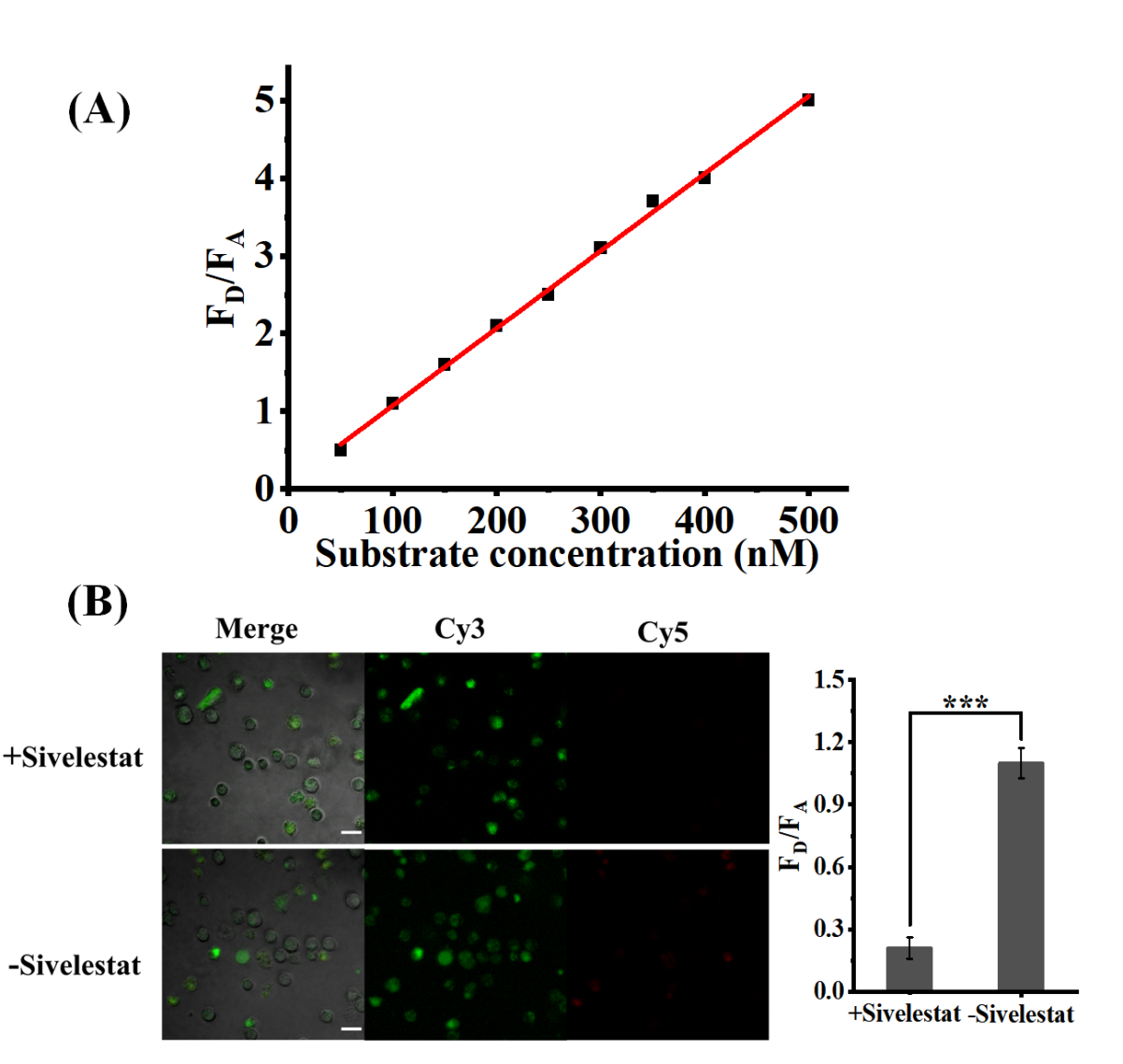


**Figure S5.** **(A)** Standard curve of F_D_/F_A_ as a function to the number of HC@Z@A/DPH nanocomposites. y = 0.00995 x + 0.07903. This curve is important to covert the F_D_/F_A_ to number of cleaved peptides and initial velocities. **(B)** Fluorescence images of THP-1 cells treated separately with 20 μM Sivelestat (top panel), and without 20 μM Sivelestat (bottom panel), followed by the incubation with HC@Z@A/DPH nanocomposites (20 μg/mL) for 2 h. Quantitative analysis of fluorescence signal changes of the left panel using the F_D_/F_A_ ratio change. *******p < 0.001.


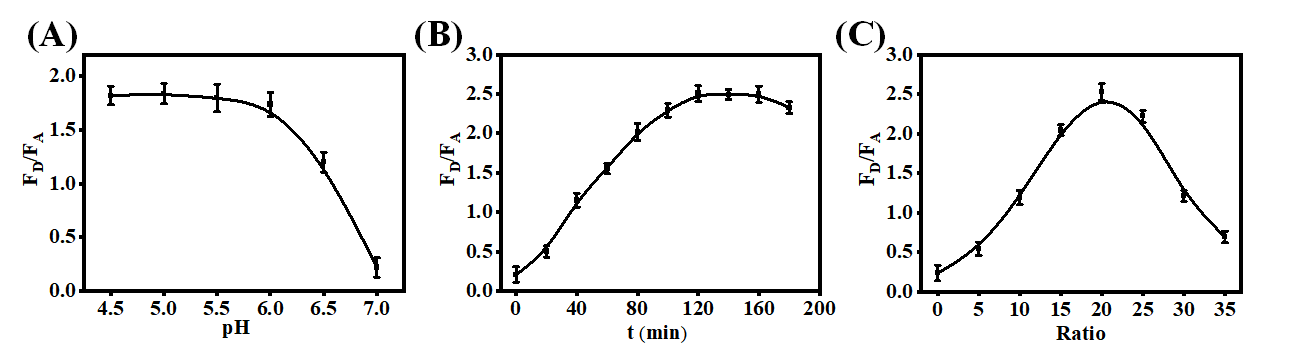


**Figure S6**. Optimization of conditions. **(A)** Effects of pH. **(B)** Entropy-driven DNA cycle circuit time. **(C)** Ratio of H3 stand to DNAzyme walking stand.


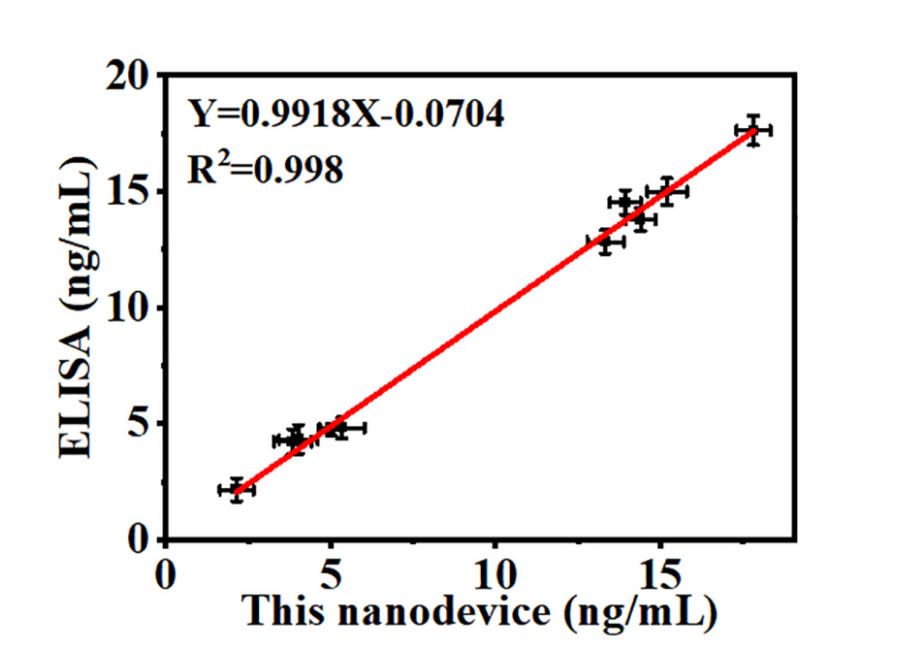


**Figure S7**. Analysis of HNE by ELISA and the proposed 3D DNAzyme motor nanodevice. Error bars show the standard deviation of three experiments.


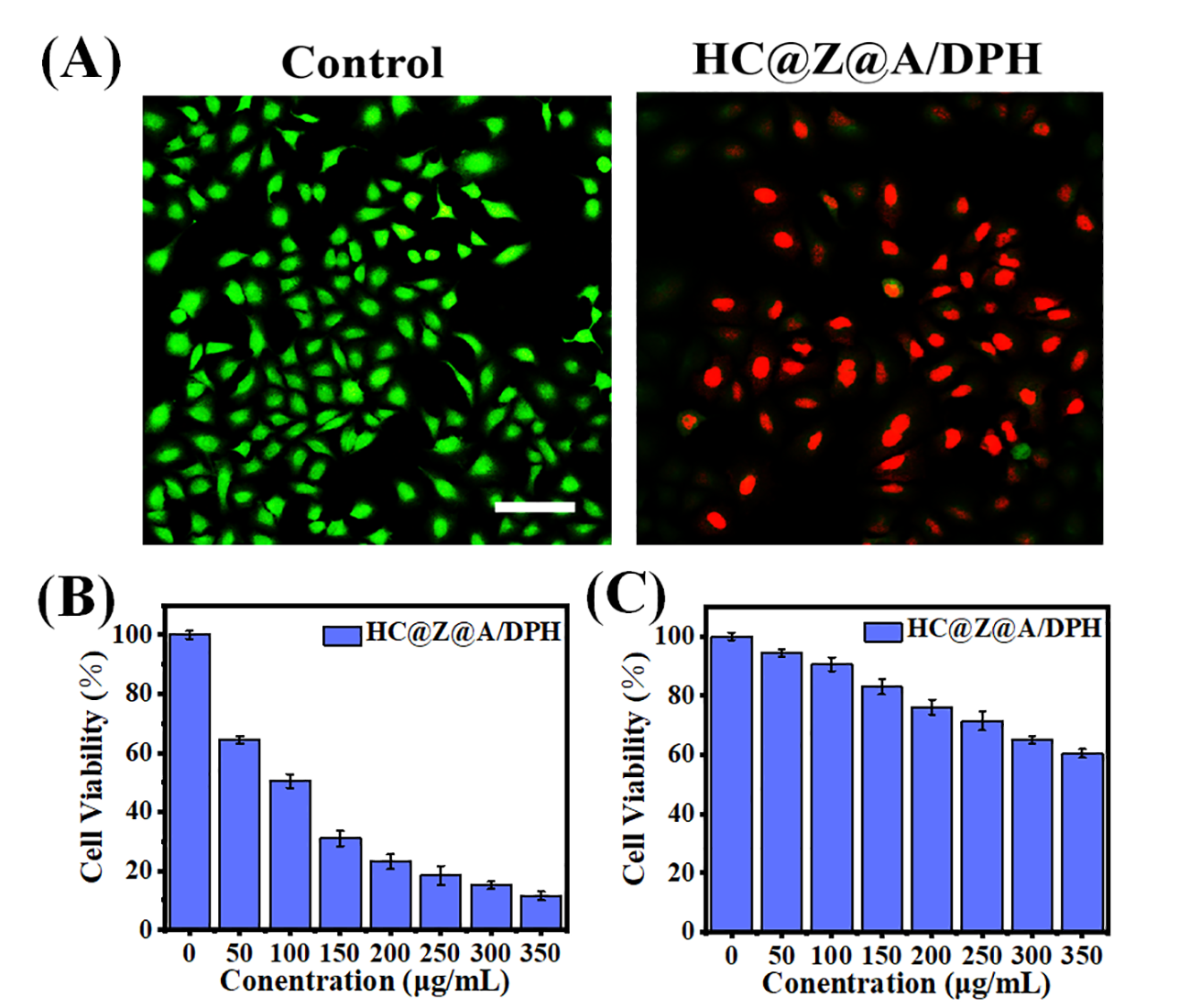


**Figure S8**. **(A)** Fluorescence images of calcein AM (green, live cells) and PI (red, dead cells) costained A549 cells after incubation with HC@Z@A/DPH nanocomposites for 24 h, the scale bar is 20 μm. **(B)** Cell viability of A549 cells after 24 h of incubation with HC@Z@A/DPH nanocomposites. **(C)** Cell viability of non-cancerous 293T cells after 24 h of incubation with HC@Z@A/DPH nanocomposites.


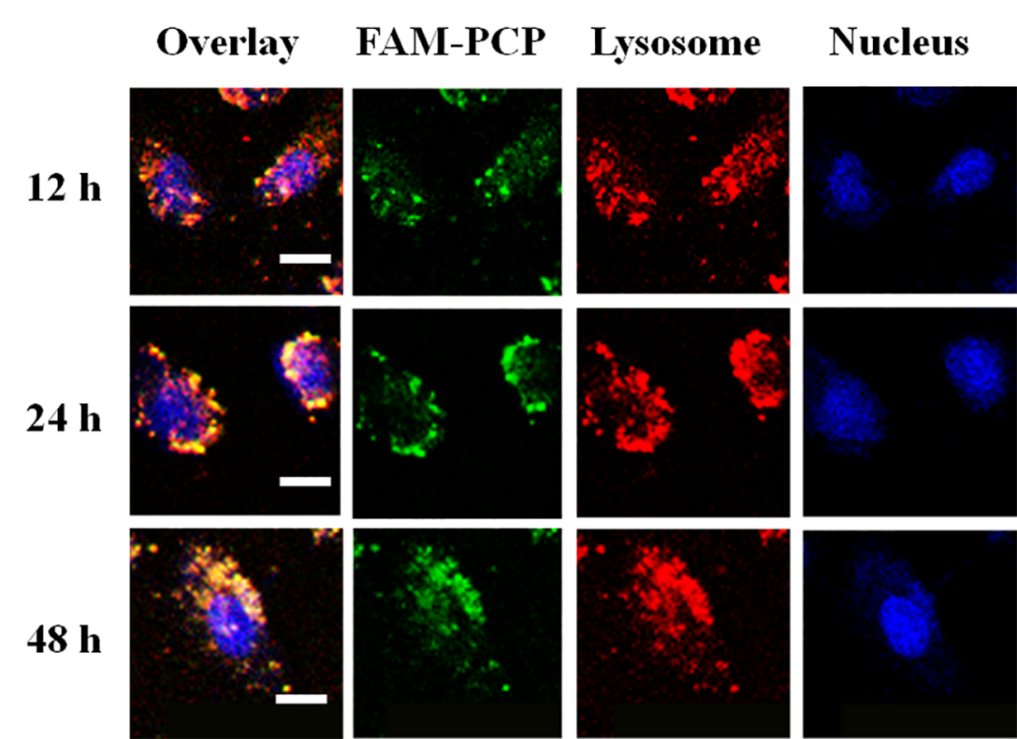


**Figure S9**. Colocalization of FAM-PCPs (green) with lysosome tracker (red) and Hoechst (nuclei, blue), the scale bar is 5 μm. The concentration of PCPs is 0.5 μg/mL, which is no obvious cytotoxicity at 0.5 μg/mL after incubation for 48 h.


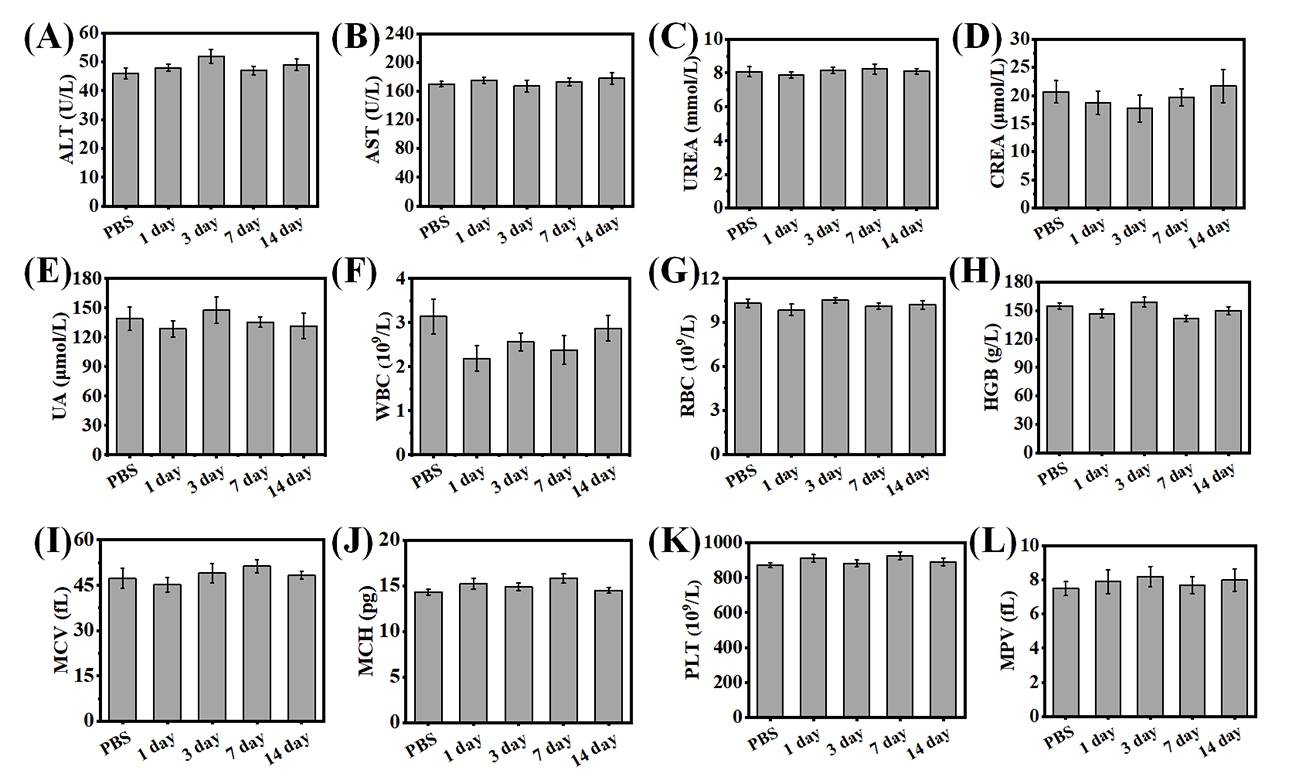


**Figure S10**. Long-term in vivo toxicity of 3D DNAzyme motor nanodevice HC@Z@A/DPH nanocomposites. **(A-E)** Blood biochemistry analyses of HC@Z@A/DPH-treated nude mice: **(A)** alanine aminotransferase (ALT), **(B)** aspartate aminotransferase (AST), **(C)** carbamide (UREA), **(D)** creatinine (CREA), and **(E)** uric acid(UA). **(F-L)** Complete blood panel analyses of these mice: **(F)** white blood cells (WBC), **(G)** red blood cells (RBC), **(H)** hemoglobin (HGB), **(I)** mean corpuscular volume (MCV), **(J)** mean corpuscular hemoglobin (MCH), **(K)** platelet (PLT), and **(L)** mean platelet volume (MPV).


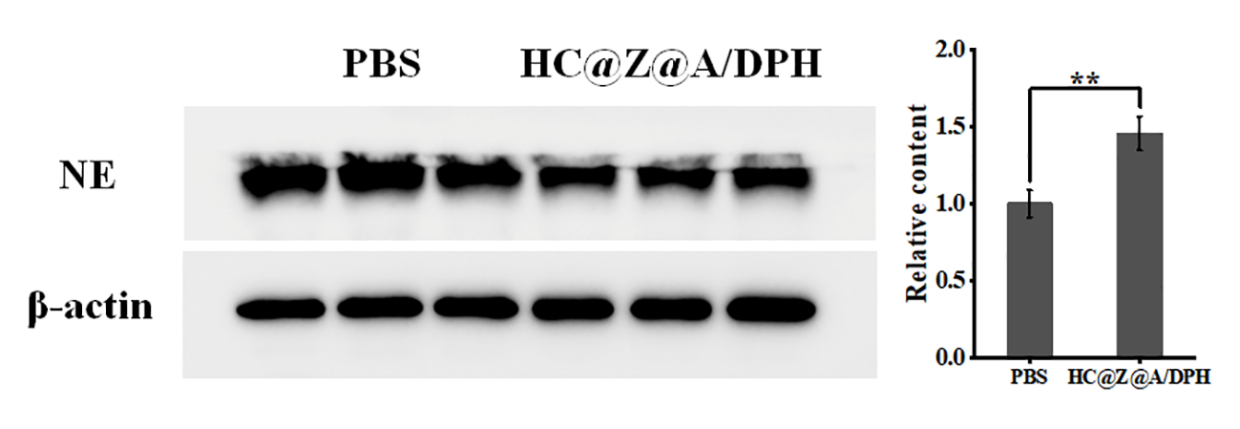


**Figure S11**. Western blot analysis of HNE expression in tumor mice before (left three bands) and after (right three bands) treatment by HC@Z@A/DPH nanocomposites, the corresponding quantitative analysis was listed in the right panel. ******p < 0.01.

| **Table S1**.The sequences of the oligonucleotides | |
| --- | --- |
| **Name** | **Sequences (5’-3’)** |
| **H1** | ATTTTTGGAGCAGGCACAACCTGCTCCAAAAATCCATT-Cy5 |
| **H2** | CATCTCTTCTCCGAGCCGGTCGTTGTGCCTGCTCCAAAAA/iCy3dT/AATGGATTTTTGGAGCAGGAAATAGTGTTT |
| **H3** | AATGGATTTTTGGAGCAGGAAACACTAT/rA/GGAAGAGATGTCCAAATATCCATT-SH |
| **DNAzyme** | SH-AAAAAAAAAAAAAAAAAAAAAAAAAAAAAAAAAAAAAAAAAAAACATCTCTTCTCCGAGCCGGTCGAAATAGTGTTTAAAAAAAAAAAAAAA-Biotin |
| **H2.1** | TTGTGCCTGCTCCAAAAA/iCy3dT/AATGGATTTTTGGAGCAGG |

| **Table S2**. Inductively coupled plasma mass spectroscopy (ICP-MS) results for Cu and CPNs content in HC@Z@A/DPH | |
| --- | --- |
| **Catalysts** | **HC@Z@A/DPH** |
| **Cu (wt％)** | **2.13** |
| **CPs (wt％)** | **3.20** |

| **Table S3**. Comparison of 3D DNAzyme motor nanodevice HC@Z@A/DPH with the reported HNE detection strategies. | | | | | |
| --- | --- | --- | --- | --- | --- |
| No. | Recognition  element | Methods | Linear range | LOD | References |
| 1 | Peptide | electrochemistry | 10-150 nM | 4 nM | Biosens. Bioelectron. 2018, 119, 209-214. |
| 2 | Peptide | Fluorescence | 1-100 ng/mL | 7.15 pM | ACS Nano 2020, 14, 4244-4254 |
| 3 | Near - infrared fluorescent organic probe | Near - infrared  fluorescence  (NIRF) and photoacoustic  (PA) imaging | 5-12.32 μM  (NIRF)  5-12.32 μM  (PA) | 0.71 nM  (NIRF)  0.67 nM  (PA) | Angew. Chem.Int. 2023, 62, e2022175 |
| 4 | Near - infrared fluorescent organic probe | Fluorescence | 0.1-1.0 μg/mL | 29.42 ng/mL | Anal. Chem. 2019, 91, 3877−3884 |
| 6 | Near - infrared fluorescent organic probe | Near - infrared  fluorescence  (NIRF) and  photoacoustic  (PA) imaging | 0.1-0.6 μg/mL  (NIRF)  0.1-0.6 μg/mL  (PA) | 15.76 ng/mL  (NIRF)  37.30 ng/mL  (PA) | Anal. Chem. 2022, 94, 3227−3234 |
| 7 | Peptide | Colorimetry | 0.025-0.2 U/mL | 0.005U/mL | Carbohydr. Polym.  2019, 216, 360-368 |
| 8 | Peptide | Fluorescence | 1-30 ng/mL | 0.026 pM | This work |
